# Monte Carlo simulation-based estimation for the minimum mortality temperature in temperature-mortality association study

**DOI:** 10.1186/s12874-017-0412-7

**Published:** 2017-09-07

**Authors:** Whanhee Lee, Ho Kim, Sunghee Hwang, Antonella Zanobetti, Joel D. Schwartz, Yeonseung Chung

**Affiliations:** 1 Department of Biostatistics and Epidemiology, Graduate School of Public Health, Seoul National University, Seoul, Republic of Korea; 2 Department of Environmental Health, Harvard T.H. Chan School of Public Health, Boston, Massachusetts USA; 3 Department of Mathematical Sciences, Korea Advanced Institute of Science and Technology, Daejeon, Republic of Korea

**Keywords:** Minimum mortality temperature, Point and interval estimation, Monte Carlo simulation-based estimation

## Abstract

**Background:**

Rich literature has reported that there exists a nonlinear association between temperature and mortality. One important feature in the temperature-mortality association is the minimum mortality temperature (MMT). The commonly used approach for estimating the MMT is to determine the MMT as the temperature at which mortality is minimized in the estimated temperature-mortality association curve. Also, an approximate bootstrap approach was proposed to calculate the standard errors and the confidence interval for the MMT. However, the statistical properties of these methods were not fully studied.

**Methods:**

Our research assessed the statistical properties of the previously proposed methods in various types of the temperature-mortality association. We also suggested an alternative approach to provide a point and an interval estimates for the MMT, which improve upon the previous approach if some prior knowledge is available on the MMT. We compare the previous and alternative methods through a simulation study and an application. In addition, as the MMT is often used as a reference temperature to calculate the cold- and heat-related relative risk (RR), we examined how the uncertainty in the MMT affects the estimation of the RRs.

**Results:**

The previously proposed method of estimating the MMT as a point (indicated as Argmin2) may increase bias or mean squared error in some types of temperature-mortality association. The approximate bootstrap method to calculate the confidence interval (indicated as Empirical1) performs properly achieving near 95% coverage but the length can be unnecessarily extremely large in some types of the association. We showed that an alternative approach (indicated as Empirical2), which can be applied if some prior knowledge is available on the MMT, works better reducing the bias and the mean squared error in point estimation and achieving near 95% coverage while shortening the length of the interval estimates.

**Conclusions:**

The Monte Carlo simulation-based approach to estimate the MMT either as a point or as an interval was shown to perform well particularly when some prior knowledge is incorporated to reduce the uncertainty. The MMT uncertainty also can affect the estimation for the MMT-referenced RR and ignoring the MMT uncertainty in the RR estimation may lead to invalid results with respect to the bias in point estimation and the coverage in interval estimation.

**Electronic supplementary material:**

The online version of this article (10.1186/s12874-017-0412-7) contains supplementary material, which is available to authorized users.

## Background

Ambient temperature has been shown to be a risk factor for mortality in numerous epidemiological studies [1–5]. Researches have reported that there exists a nonlinear association between temperature and mortality, characterized by U- or J- shaped association [1–3]. One important feature in the temperature-mortality association is the minimum mortality temperature (MMT), which is defined as the temperature at which the lowest mortality is achieved. The MMT has been regarded as a threshold point in describing the population susceptibility to temperature [5] as mortality increases with temperature increasing or decreasing from the MMT. Therefore, the MMT is often used as a reference temperature to quantify the relative risk (RR) related to cold or hot temperatures in many previous studies [1, 5].

Despite the importance of the MMT, little research has been conducted on statistical inference on the MMT. A recently proposed approach for estimating the MMT in a nonlinear temperature-mortality association is to determine the MMT as the temperature at which mortality is minimized in the estimated temperature-mortality association curve [1, 5]. This approach provides a point estimate but the corresponding uncertainty is not quantified. Another study [6] proposed an approximate bootstrap approach to calculate the standard errors and the confidence interval for the MMT. The study applied the method to the data for 52 cities in Spain and showed that the uncertainty can be small or large depending on the estimated association pattern which varies among cities.

The statistical properties of the previously proposed methods were not fully studied. Our research aims to assess these methods in various types of the temperature-mortality association via a simulation study. Then, we suggest an alternative approach to provide a point and an interval estimates for the MMT, which may improve upon the previous approach if some prior knowledge is incorporated for the potential range of the MMT. We compare the previous and alternative methods through a simulation study and an application. Additionally, as the MMT is often used as a reference temperature to calculate the cold- and heat-related RRs [1], we assess how the uncertainty in the MMT affects the estimation of the RRs.

In Methods section, we describe (1) how we model the temperature-mortality association, (2) the previous and alternative methods to calculate a point and an interval estimates for the MMT, (3) the design of the simulation study, and (4) the modeling details of the US data analysis. In Results section, we report the results from the simulation study and the data analysis for the 135 US cities. We included discussions and conclusions in the two final sections.

## Methods

### Modeling the temperature-mortality association

Let *Y*_*t*_ be the daily death count on day t, with t = 1 , … , *N*, and **x**_*t*_ = (*x*_*t*_, *x*_*t* − 1_, …, *x*_*t* − *L*_)^′^ be the vector of daily mean temperatures on day t and over the previous L days. We model the association between *Y*_*t*_ and **x**_*t*_ using a generalized linear model (GLM) with a quasi-Poisson family.

$$ {Y}_t\sim Quasi- Poisson\left({\mu}_t\right) $$

1 $$ \log \left({\mu}_t\right)=\alpha +s\left({\mathbf{x}}_t;\boldsymbol{\eta} \right)+{\sum}_{j=1}^J{h}_j\left({u}_{jt};{\boldsymbol{\gamma}}_j\right) $$

where *μ*_*t*_ is the expected death count on day t, s(·) is a flexible function characterized by parameter ***η*** to depict the effects of temperature, *u*_*jt*_ is the j-th confounding variable measured on day t, *h*_*j*_(·) is a flexible function to represent the effects of *j*-th confounding variable, and ***γ***_*j*_ is the corresponding parameter. We assume a quasi-Poisson family to allow for overdispersion (meaning that the variance of the outcome counts is higher than predicted under a Poisson distribution) which is a well-known feature observed in the time-series analysis for temperature-mortality association [7]. For s(), we use the distributed lag nonlinear model (DLNM) [7] to describe the nonlinear and lagged dependency as has been used in many of the previous studies [1, 2, 5–8]. In DLNM, a cross-basis is specified for temperature and lag. Let ${\phi}_1\left(\cdotp \right),\cdots, {\phi}_{v_x}\left(\cdotp \right)$ be the basis to describe the temperature-mortality association and ${\psi}_1\left(\cdotp \right),\cdots, {\psi}_{v_l}\left(\cdotp \right)$ be the basis to depict the lag-mortality association. The DLNM is expressed as.

2 $$ s\left({\mathbf{x}}_t;\boldsymbol{\eta} \right)={\sum}_{j=1}^{v_x}{\sum}_{k=1}^{v_l}{\mathbf{r}}_{tj}^{\prime }{\mathbf{c}}_k{\eta}_{jk} $$

where **r**_*tj*_ = (*ϕ*_*j*_(*x*_*t*_),  ⋯ , *ϕ*_*j*_(*x*_*t* − *L*_))^′^ is the vector of **x**_*t*_ transformed through the *j*-th basis *ϕ*_*j*_(·) in the temperature dimension and **c**_*k*_ = (*ψ*_*k*_(0),  ⋯ , *ψ*_*k*_(*L*))^′^ is the vector derived by applying the *k*-th basis *ψ*_*k*_(·) for lag dimension to the vector (0,  ⋯ , *L*)^′^. Then, $\boldsymbol{\eta} ={\left({\eta}_{11},\dots, {\eta}_{v_x{v}_l}\right)}^{\prime }$ is the vector of coefficients for the cross-basis with the dimension *v*_*x*_ × *v*_*l*_. Different choices of the basis can be considered in DLNM and model selection criteria such as QAIC or QBIC (Akaike and Bayesian information criteria for models with overdispersed outcomes fitted through quasi-likelihood) can be used to determine an optimal choice [8].

In order to estimate the lag-cumulated temperature-mortality association, ***η*** is reduced through the following transformation [9].

$$ \beta =\mathrm{M}\eta $$

3 $$ \mathrm{V}\left(\beta \right)=\mathrm{M}\ V\left(\eta \right)\ {\mathrm{M}}^T $$

where $\mathbf{M}={1}_{\left(L+1\right)}^{\prime}\mathbf{C}\bigotimes {\mathbf{I}}_{\left({v}_x\right)}$ is a reducing matrix, $\boldsymbol{\beta} ={\left({\beta}_1,{\beta}_2,\dots, {\beta}_{v_x}\right)}^{\prime }$ is the reduced parameter, and *V*(***β***) is the associated error (co)variance matrix. In **M,**

4 $$ \mathrm{C}=\left({c}_1,\cdots, {\mathrm{c}}_{v_l}\right)=\left(\begin{array}{ccc}\hfill \begin{array}{cc}\hfill {\psi}_1(0)\hfill & \hfill {\psi}_2(0)\hfill \\ {}\hfill {\psi}_1(1)\hfill & \hfill {\psi}_2(1)\hfill \end{array}\hfill & \hfill \begin{array}{c}\hfill \cdots \hfill \\ {}\hfill \cdots \hfill \end{array}\hfill & \hfill \begin{array}{c}\hfill {\psi}_{v_l}(0)\hfill \\ {}\hfill {\psi}_{v_l}(1)\hfill \end{array}\hfill \\ {}\hfill \vdots \vdots \hfill & \hfill \ddots \hfill & \hfill \vdots \hfill \\ {}\hfill {\psi}_1(L)\kern0.5em {\psi}_2(L)\hfill & \hfill \cdots \hfill & \hfill {\psi}_{v_l}(L)\hfill \end{array}\right) $$

and ⨂ is the notation of the Kronecker product. Then, ***β*** is the parameter to describe the temperature-mortality association cumulated over the lags.

### Estimating the minimum mortality temperature (MMT)

First, we describe the previously proposed approach to estimate the MMT [6]. Let $\widehat{\ \boldsymbol{\beta}}$ be the maximum likelihood estimate obtained from model (1) through (3). Given $\widehat{\ \boldsymbol{\beta}}$, the previously proposed point estimate for the MMT is a solution of $\underset{x}{\mathrm{argmin}\Big(}{\boldsymbol{Q}}_x\widehat{\boldsymbol{\beta}}\Big)$ where ${\boldsymbol{Q}}_x=\left({\phi}_1(x),\cdots, {\phi}_{v_x}(x)\right)$is the vector of basis variables by applying the basis for temperature to a particular temperature value *x*, and *x* ranges from the minimum to maximum temperatures observed in the data. The solution can be the minimum or maximum temperature, in which case it has been suggested to constrain the solution within the 1st – 99th percentiles of the temperature. To quantify the uncertainty, an approximate bootstrap method was proposed to derive the empirical distribution of the MMT. Based on the maximum likelihood principle [10], if the sample size is sufficiently large, it can be assumed that the true **β** follows a multivariate normal distribution with the mean as the estimate ($\widehat{\boldsymbol{\beta}}$) and the variance as the corresponding error (co)variance ($\mathrm{V}\left(\widehat{\boldsymbol{\beta}}\right)$) [11–13]. Then, one can simulate the true **β** and the true MMT through the following procedure.

$$ sample\ {\boldsymbol{\beta}}_{(i)}\sim MVN\left(\widehat{\boldsymbol{\beta}},\mathrm{V}\left(\widehat{\boldsymbol{\beta}}\right)\right) $$

5 $$ {\theta}_{(i)}=\underset{x}{\mathrm{argmin}\Big(}{\boldsymbol{Q}}_x{\boldsymbol{\beta}}_{(i)}\Big) $$

where (*i*) indicates i-th simulated sample, ***β***_(*i*)_ are independent and identically distributed sample, and *θ*_(*i*)_ are the samples to approximate the empirical distribution of the true MMT. Then, based on the empirical distribution of the MMT, it was proposed to use the empirical percentiles (i.2., 2.5th - 97.5th) as an interval estimate for the MMT (i.e., 95% confidence interval (CI)).

Now, we describe an alternative procedure to estimate the MMT, which may improve upon the previous method when a prior knowledge is available on the MMT. In the previous approach, the empirical distribution for the MMT is determined by the multivariate normal distribution with mean ($\widehat{\boldsymbol{\beta}}$) and (co)variance ($\mathrm{V}\left(\widehat{\boldsymbol{\beta}}\right)$), and thus the uncertainty for the MMT tends to be large if $\mathrm{V}\left(\widehat{\boldsymbol{\beta}}\right)$is large. In such case, adding some restrictions for the MMT distribution based on a prior knowledge may reduce the uncertainty. Applying a Bayesian inferential framework, we specify a prior distribution for the MMT and combine it with the sampling procedure (5). That way, a posterior distribution for the MMT is derived as a trade-off between the prior knowledge and the information in the data. In the context of the MMT, a realistic prior would be a Uniform distribution with a support (*α*_1_ , *α*_2_) representing a plausible range of the MMT. The support can vary depending on the level of informativity of prior knowledge (e.g., minimally informative prior range: 1st – 99th percentiles of observed temperature distribution or strongly informative range: 50th -70th percentiles). With such prior assumption, the posterior distribution can be obtained through the sampling procedure (5) by discarding the samples of *θ*_(*i*)_ which do not fall within the range of (*α*_1_,*α*_2_). That is,

$$ sample{\boldsymbol{\beta}}_{(i)} suchthat{\alpha}_1\le \underset{x}{\mathrm{argmin}\Big(}{\boldsymbol{Q}}_x{\boldsymbol{\beta}}_{(i)}\Big)\le {\alpha}_2 $$

6 $$ {\theta}_{(i)}=\underset{x}{\mathrm{argmin}\Big(}{\boldsymbol{Q}}_x{\boldsymbol{\beta}}_{\left(\boldsymbol{i}\right)}\Big) $$

Then, the empirical mean (or median) and percentiles (e.g., 2.5th - 97.5th) can serve as a point and an interval estimates for the MMT. The empirical distribution of the MMT is often not symmetric but skewed, and in such case, the choice of percentiles may be adjusted depending on the shape of the empirical distribution (e.g., 0th – 95th percentiles for a highly right-skewed case).

### Estimating the relative risk (RR) accounting for the uncertainty of MMT

Here, we describe how we estimate an RR with the MMT used as a reference temperature accounting for the uncertainty in the MMT. Given the Monte Carlo samples of ***β***_(*i*)_ and *θ*_(*i*)_ obtained through procedure (5) or (6), one can calculate an RR comparing an arbitrary temperature value *x* and the MMT as

$$ {\boldsymbol{\zeta}}_{(i)}=\left({\boldsymbol{Q}}_x-{\boldsymbol{Q}}_{\theta_{(i)}}\right){\boldsymbol{\beta}}_{(i)} $$

7 $$ \exp \left({\boldsymbol{\zeta}}_{(i)}\right)={\boldsymbol{RR}}_{(i)} $$

where ***ζ***_(*i*)_ indicates the log of RR calculated using i-th sample of ***β***_(*i*)_ and *θ*_(*i*)_ and ***RR***_(*i*)_ is the i-th sample of the true RR. Then, a point and an interval estimates for the RR can be derived from the empirical distribution of the RR in the same way as the MMT estimates. Often, scientific interest is on the cold- and the heat- related RRs which are defined as the RRs comparing the 1st percentile of temperature distribution and the MMT and comparing the 99th percentile and the MMT, respectively. Hereafter, we call these RRs as the cold- and heat- related RRs.

### Simulation study

Simulation study was carried out to compare different methods in estimating the MMT and the cold- and heat-related RRs. We considered six methods. The first one (named as Argmin1) is to use the solution of the $\underset{x}{\mathrm{argmin}\Big(}{\boldsymbol{Q}}_x\widehat{\boldsymbol{\beta}}\Big)$ without any constraint as a point estimate for the MMT and to use the MMT estimate for calculating the RRs. The second method (named as Argmin2) is the same as the first one except that the solution is constrained within the 1st - 99th temperature percentiles. The third method (named as Empirical1) is to use the empirical mean and percentiles (2.5th – 97.5th) as a point and an interval estimates for the MMT without any prior knowledge combined, and to calculate the RR accouting for the MMT uncertainty. The fourth, fifth, and sixth methods (named as Empirical2_strong_, Empirical2_moderate_, and Empirical2_minimal_) are the same as the third one except that the empirical distribution of MMT is derived with prior knowledge. Empirical2_strong_, Empirical2_moderate_, and Empirical2_minimal_ incorporate strongly, moderatly, and minimally informative priors, respectively.

To generate the data, four different scenarios were considered for the temperature-mortality association: U-shape (Scenario 1), reverse J-shape (Scenario 2), rotated S-shape (Scenario 3) and sector shape (Scenario 4). Additional file 1: Figure S1 displays the shape of the true RR curve and the true MMT. To obtain the model parameters for each scenario, we used part of the US data analyzed in the application section. For scenarios 1, 2, and 4, we fit eqation (1) for the data of New York with temperature metric as 0–2 day moving average, 0–1 day moving average, and the current day value, respectively. For scenario 3, the same model was fit with 0–3 day moving average for the data of Ockland. For all scenarios, we controlled for the day of week using indicator variables and for the long-term and seasonal pattern using natural cubic spline with 8 degree of freedom for each year. For s(·), as we use moving average as temperature metric, we used one-dimensional basis (quadratic B-spline with the knots placed at the 10th, 75th, and 90th percentiles). Once the parameters are estimated, the mortality data were generated from the fitted model using the covariates in the data for each scenario. For the distribution for mortality, we considered Quasi-Poisson distribution with the overdispersion parameter set to be equal to the model fit.

For each scenario, we generated 1000 replicates of dataset. For each dataset, we fitted eq. (1) with the same specifications used to generate the data and obtained the coefficient estimates. Because we use moving avearage as temperature metric, which is a special case of distributed lag nonlineear model, the coefficients in eq. (1) can be considered as the reduced coefficients (***β***) in eq. (3). Using the coefficient estimates, we estimated the MMT and the cold- and heat-related RRs by the six different methods. For Empirical2, we incorporated prior knowledge with different levels of informativity using Uniform prior with different supports. Empirical2_strong_ uses, as the prior support, the 70th - 95th temperature percentiles for scenarios 1 and 3, the 40th - 65th for scenario 2, and the 1st - 10th for scenario 4. Empirical2_moderate_ uses the 50th -99th percentiles for scenario 1 and 3, the 30th – 80th for scenario 2, and the 1st – 50th for scenario 3. Empirical2_minimal_ uses the 1st -99th percentiles for all scenarios. These prior ranges are indicated in Additional file 1: Figure S1. To compare different methods, we calculated mean bias (Bias) and root mean squared error (RMSE) for the point estimate and coverage probability (%CP) and mean length (Length) of the interval estimate for the MMT and the cold- and heat-related RRs using the 1000 replicates of dataset.

Additionally, we conducted a series of sensitivity analysis to evaluate the robustness of different methods varying the sample size and the specification of the splines and knots in modeling the temperature-mortality association. We considered five methods excluding Empirical2_moderate_ as its performance is between Empirical2_strong_ and Empirical2_minimal_. First, we varied the sample size, 5 and 10 years of data, and compare with the full period (22 years) of data. Second, we varied the splines, natural cubic B-splines and quadratic B-splines, in the true and fitted models. Finally, we varied the locations of the knots, a set of 25th, 50th, and 75th temperature percentiles and another set of 10th, 75th, and 95th percentiles, in the true and fitted models.

### Application

We applied three methods (Argmin2, Empirical1, and Empirical2_minimal_) to estimate the MMT and the cold- and heat-related RRs in the temperature-mortality association for 135 cities in the US for the period of January 1, 1985 to December 31, 2006. Daily mortality counts were obtained from the National Center for Health Statistics and non-external cause mortality counts were used (ICD-9: 0–799; ICD-10: A00–R99). Daily mean temperatures (24-h mean) were obtained from the National Climate Data Center of the National Oceanic and Atmospheric Administration. These data were analyzed in a previous study [1] and the city-specific descriptive statistics are reported in Additional file 1: Table S1.

For each city, we fit eqs. (1) and (2) with the following modeling choices. For cross-basis, the quadratic B-spline was used with the knots placed at the 10th, 75th, and 90th percentiles of the city-specific temperature distributions. For the lagged dependency, we used the natural cubic B-spline with an intercept and three internal knots (equally spaced values in the log scale) with 21 lag days. We controlled for the day of week using indicator variables and for the seasonal and long-term trends via a natural cubic B-spline of time with 8 degrees of freedom per year. These choices were based on the results in a previous study [1]. Because the city-specific modeling accompanies relatively large estimation error, we combined evidence across all cities using multivariate meta-regression [14] with city-specific average temperature and temperate range as meta-predictors and obtained the best linear unbiased predictor (BLUP) $\widehat{\boldsymbol{\beta}}$ and the corresponding standard error for each city. Then, using the BLUPs**,** we applied the three methods for estimating the MMT and the cold- and heat- related RRs. For Empirical2_minimal_, we assumed Uniform prior with the support as the 1st - 99th percentiles of city-specific temperature.

## Results

### Simulation study

Table 1 reports the results in estimating MMT by six different methods. Because Argmin1 and Argmin2 do not provide an interval estimate, the %CP and Length are not reported. In scenario 1, all six methods show small Bias and small RMSE. In scenarios 2–4, Argmin1 and Empirical1 show relatively large Bias and large RMSE (8.896 and 6.979 in scenario 2, 28.011 and 21.092 in scenario 3, and 8.686 and 6.259 in scenario 4) while Argmin2 and Empirical2’s show small Bias and small RMSE (mostly less than 3 and 3.592 at maximum). The %CP is near or greater than 95% for Empirical1 and Empirical2’s in all scenarios except that the %CP was relatively lower for Empirical2_moderate_ and Empirical2_minimal_ in scenario 4. In scenario 4, the empirical distribution of the MMT was observed as highly right-skewed with the two empirical methods and an adjustment to percentiles in the CI as the 0th – 95th recovered over 95% coverage (98.6% and 98.3%, respectively). The Length was similar between Empirical1 and Empirical2_minimal_ and smaller for Empirical2_moderate_ and Empirical2_strong_ in all scenarios. In summary, our results suggest that Argmin2 (previously proposed in [6]) is a reasonable point estimator, though RMSE may increase depending on the association shape, and Empirical1 (previously proposed in [6]) provides an interval estimate with near 95% coverage while the length can be too large in some scenarios. If prior knowledge is available even at a minimal level, the RMSE can be reduced with Empirical2’s compared with Argmin2, and the Length of the interval estimate can be shorter with Empirical2’s compared with Empirical1 still achieving the similar level (near 95%) of coverage probability.

**Table 1:** Mean Bias (Bias) and root mean squared error (RMSE) for the point estimate and the coverage probability (% CP) and mean length (Length) of the interval estimate in estimating the minimum mortality temperature (MMT) by six different methods (Argmin1, Argmin2, Empirical1, Empirical2_strong_, Empirical2_moderate_, and Empirical2_minimal_) for each of the 4 scenarios; U-shape (Scenario 1), reverse J-shape (Scenario 2), rotated S-shape (Scenario 3) and sector shape (Scenario 4)

		Methods					
		Argmin 1	Argmin 2	Empirical 1	Empirical 2_strong_^a^	Empirical 2_moderate_^b^	Empirical 2_minimal_^c^
Scenario 1 (True MMT = 23.889)	Bias	-0.178	-0.183	-0.203	-0.203	-0.194	-0.152
	RMSE	1.046	1.073	0.859	0.836	0.823	0.870
	% CP			97.2%	96.4%	96.8%	95.6%
	Length			3.385	3.210	3.410	3.387
Scenario 2 (True MMT = 11.274)	Bias	2.680	-0.245	4.197	-1.183	-0.772	-0.311
	RMSE	8.896	2.243	6.979	1.542	2.279	2.428
	% CP			96.4%	98.0%	95.3%	93.8%
	Length			10.415	5.631	8.917	10.440
Scenario 3 (True MMT = 29.167)	Bias	16.486	0.512	16.683	0.776	0.639	1.240
	RMSE	28.011	3.342	21.092	1.126	1.354	2.385
	% CP			96.5%	96.4%	95.9%	96.5%
	Length			9.758	3.257	5.662	9.054
Scenario 4 (True MMT = −3.333)	Bias	4.359	0.099	4.340	-1.069	-2.201	-2.181
	RMSE	8.686	3.592	6.259	1.504	2.815	2.835
	% CP			95.4%	93.8%	85.9%	84.7%
	Length			7.816	6.097	7.719	7.681

^a^Prior support: 70th -95th percentiles for scenarios 1 & 3, 40th – 65th percentiles for scenario 2, and 1st -10th percentiles for scenario 4

^**b**^Prior support: 50th -99th percentiles for scenarios 1 & 3, 30th -80th percentiles for scenario 2, and 1st -50th percentiles for scenario 4

^c^Prior support: 1st – 99th percentiles for all scenarios

Table 2 reports the results in estimating the cold- and heat-related RRs by six different methods. For cold-related RR, both Bias and RMSE were small and the %CP was near 95% in scenario 1 and 2. In scenario 3, RMSE was relatively large and the %CP was low as 86.8% with Empirical1 but near 95% with other methods. In scenario 4, both Bias and RMSE was small but the %CP was low as 58.4%, 70.3%, 76.5%, and 75.6% with Argmin2, Empirical1, Empirical2_moderate_ and Empirical2_minimal_. For heat-related RR, both Bias and RMSE were small and the %CP was near 95% in scenario 1 and 2. In scenario 3, RMSE was relatively large and the %CP was low as 85.8% and 89.2% with Argmin2 and Empirical1 but near 95% with Argmin1 and Empirical2. In scenario 4, RMSE was somewhat large and the %CP was low as 90% with Empirical1 but near 95% with other methods. In summary, in estimating the RR, Argmin1 seems to result in an appropriate coverage but may lead to large RMSE while Argmin2 may result in low coverage but small RMSE in various scenarios. Empirical1 can result in low coverage and large RMSE. However, Empirical2’s were shown to perform well generally in various scenarios in both aspects of RMSE and coverage even with minimally informative priors.

**Table 2:** Mean Bias (Bias) and root mean squared error (RMSE) for the point estimate and the coverage probability (%CP) of the interval estimate in estimating the cold- and heat-related relative risk (RR) by six different methods (Argmin1, Argmin2, Empirical1, Empirical2_strong_, Empirical2_moderate_, and Empirical2_minimal_) for each of the 4 scenarios; U-shape (Scenario 1), reverse J-shape (Scenario 2), rotated S-shape (Scenario 3) and sector shape (Scenario 4)

			Methods					
			Argmin1	Argmin2	Empirical1	Empirical2_strong_^a^	Empirical2_moderate_^b^	Empirical2_minimal_^c^
Cold-related RR	Scenario 1 (True RR = 1.094)	Bias	-0.0002	0.0001	-0.0009	-0.0008	-0.0003	-0.0006
		RMSE	0.0093	0.0090	0.0094	0.0092	0.0090	0.0090
		% CP	94.2%	95.6%	94.9%	96.7%	95.2%	95.0%
	Scenario 2 (True RR = 1.023)	Bias	-0.001	-0.0004	-0.004	-0.006	-0.0038	-0.0030
		RMSE	0.0069	0.0067	0.0073	0.0069	0.0056	0.0061
		% CP	95.4%	94.0%	95.7%	96.2%	96.7%	95.4%
	Scenario 3 (True RR = 1.070)	Bias	-0.030	-0.005	-0.046	-0.022	-0.280	-0.0221
		RMSE	0.0548	0.0287	0.0548	0.0250	0.0325	0.0275
		% CP	94.6%	93.5%	86.8%	99.7%	96.0%	98.0%
	Scenario 4 (True RR = 1.000)	Bias	-0.008	-0.002	-0.013	-0.005	-0.0059	-0.0061
		RMSE	0.0126	0.0036	0.0155	0.0055	0.0061	0.0063
		% CP	95.8%	58.4%	70.3%	93.2%	76.5%	75.6%
Heat-related RR	Scenario 1 (True RR = 1.079)	Bias	-0.0005	-0.0005	-0.0006	-0.001	-0.0007	-0.0011
		RMSE	0.0062	0.0064	0.0062	0.0062	0.0063	0.0061
		% CP	96.0%	95.4%	95.5%	93.5%	93.9%	95.8%
	Scenario 2 (True RR = 1.118)	Bias	-0.002	0.0001	-0.004	0.0005	0.0003	-0.0005
		RMSE	0.0100	0.0084	0.0114	0.0050	0.0068	0.0075
		% CP	95.6%	95.0%	94.3%	99.9%	99.5%	98.8%
	Scenario 3 (True RR = 1.015)	Bias	-0.027	-0.003	-0.044	-0.019	-0.0122	-0.121
		RMSE	0.0548	0.0144	0.0632	0.0192	0.0145	0.0144
		% CP	95.6%	85.8%	89.2%	98.1%	99.0%	98.8%
	Scenario 4 (True RR = 1.185)	Bias	-0.010	-0.0016	-0.016	-0.006	-0.0023	-0.0019
		RMSE	0.0205	0.0111	0.0232	0.0085	0.0079	0.0073
		% CP	96.2%	95.1%	90.0%	99.4%	99.6%	100%

^a^Prior support: 70th -95th percentiles for scenarios 1 & 3, 40th – 65th percentiles for scenario 2, and 1st -10th percentiles for scenario 4

^b^Prior support: 50th -99th percentiles for scenarios 1 & 3, 30th -80th percentiles for scenario 2, and 1st -50th percentiles for scenario 4

^c^Prior support: 1st – 99th percentiles for all scenarios

Additional file 1 Figures S2-S7 report the results of the sensitivity analysis. Additional file 1: Figures S2 & S3 show the RMSE and %CP in estimating the MMT with varying sample size, Additional file 1: Figures S4 & S5 with different specifications of the splines, and Additional file 1: Figures S6 & S7 with different specifications of the knots. Additional file 1: Figure S2 shows that Empirical2_minimal_ and Empirical2_stong_ show lowest RMSE among other methods and stable RMSE over different sample sizes in all scenarios. Additional file 1: Figure S3 shows the %CP was comparable among Empirical1, Empirical2_minimal_, and Empirical2_strong_ in all scenarios with an exception of scenario 4 where Empirical2_minimal_ shows slightly lower coverage for all sample sizes. As mentioned earlier, the coverage was recovered with an adjustment to the percentiles to obtain the CI. Additional file 1: Figure S4 indicates that the RMSE with Empirical2’s was lowest and stable over different choices of splines in the true and fitted models. Additional file 1: Figure S5 shows that the %CP was generally comparable although the coverage reduced significantly with Empirical2’s when the splines are miss-specified (case 3 in scenario 1 and case 2 in scenario 3). In those cases, the CIs tended to miss the true MMT right above the upper bound due to the very short length. Finally, Additional file 1: Figure S6 displays that the RMSE was lowest and stable over different specifications of the knots in the true and fitted models. However, Additional file 1: Figure S7 shows that the miss-specifications of the knots resulted in reducing the coverage in case 2 for scenario 1 and 3. Similarly, the CIs tended to miss the true MMT right above the lower bound

### Application

**Fig. 1:**
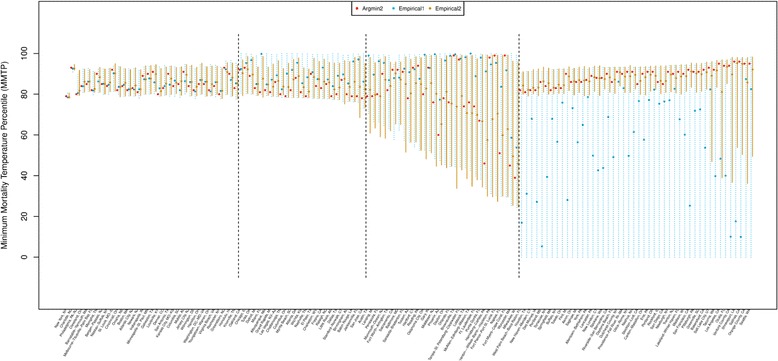
Estimated minimum mortality temperature (MMT) percentile for 135 cities in the US by three different methods; Argmin2 (*red*), Empirical1 (*blue*), Empirical2_minimal_ (*orange)*. Points indicate the point estimate and vertical solid/dashed bars indicate 95% empirical interval estimates. Cities are ordered according to the MMT uncertainty (the length of the interval estimates obtained by Empirical1). The cities are divided into 4 categories (indicated by *black* dashed vertical lines) with respect to the MMT uncertainty and temperature-mortality association types (refer to Fig. S8)

Figure 1 displays the point estimates for the MMT obtained by Argmin2, Empirical1, and Empirical2_minimal_ and the interval estimates by the two empirical methods. The cities are ordered based on the length of the interval estimate calculated by Empirical1. Based on the MMT uncertainty patterns, it seems reasonable that we divide 135 cities into four categories; category 1 (from New York to Knoxville), category 2 (from Oakland to San Jose), category 3 (from Austin to Milwaukee), and category 4 (from Western Palm Beach-Boca Raton to Seattle). Such categorization also consists with the shapes of the RR curve for each city presented in Additional file 1: Figure S8 (The cities are in the same order as in Fig. 1). In category 1, cities show clear U-shape RR curve and small uncertainty in the MMT. Accordingly, the point estimates were close among the three methods and the interval estimates were similar between the two empirical methods. In category 2, cities show U-shape RR curve with a short right arm and a short bottom and small uncertainty in the MMT. Argmin2 tends to suggest a smaller value for the MMT estimate, Empirical1 suggests larger point estimate, and Empirical2_minimal_ compromises between the Argmin2 and Empirical1 estimates. In category 3, the RR curve is mostly reverse J-shape or U-shape with a wide bottom and the MMT uncertainty become large. For the reverse J-shape curve, Empirical1 suggests the largest value for the MMT while Argmin2 and Empirical2_minimal_ suggests somewhat smaller values. In category 4, cities show rotated S-shape or widely opened U-shape with large uncertainty on both arms. Empirical1 suggests extremely large uncertainty in the MMT covering almost the whole range of the temperature distribution. However, the uncertainty reduced largely by Empirical2_minimal_ with the prior restriction, within the 1st – 99th percentiles.

**Fig. 2:**
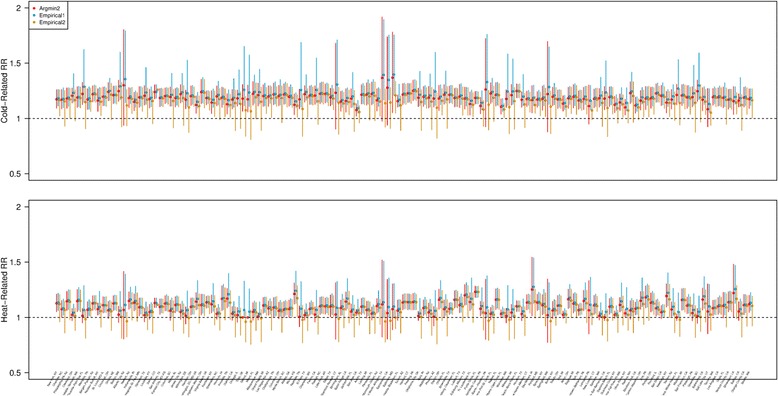
Estimated cold- and heat- related relative risk (RR) for 135 cities in the US by three different methods; Argmin2 (*red*), Empirical1 (*blue*), Empirical2_minimal_ (*orange*). Points indicate the point estimate and vertical solid/dashed bars indicate 95% empirical interval estimates. Cities are ordered according to the MMT uncertainty (the length of the interval estimates obtained by Empirical1) as in Fig. 1

Figure 2 shows the point and interval estimates for the cold-related and heat-related RRs calculated by the three methods. Differently from the MMT estimates, the point estimates were mostly consistent among the three methods. However, the intervals estimates calculated by Argmin2 and Empirical1 tend to stretch to the right while those computed by Empirical2_minimal_ tend to do to the left. Such result is consistent with simulation results in that the coverage for the RR were low with Argmin2 and Empirical1 as those intervals tend to exclude the true RR because of the right-skewness of the empirical distribution.

## Discussion

In this research, we assessed the statistical properties of the previously proposed statistical approach [6] to estimate the MMT in various types of association via a simulation study and an application. The method of using the solution of argmin function with some ad hoc restriction (i.e., within the 1st – 99th percentiles of the observed temperature distribution) (Argmin2) turns out to be a reasonable point estimator for the MMT, though Bias or RMSE may be large in some scenarios. Also, the approximate bootstrap method to calculate the confidence interval (Empirical1) performs properly achieving near 95% coverage, though the length can be extremely large depending on the scenarios.

To improve upon the previous method, we suggested an alternative approach (Empirical2), which can be applied if some prior knowledge is available on the MMT. We suggested to combine a prior knowledge with the procedure of deriving the empirical distribution of the MMT and to use the empirical mean and percentiles (e.g., 2.5th - 97.5th in general and 0th – 95th for highly right-skewed case) as a point and an interval estimates for the MMT. Simulation study showed that our proposed method performs better even with a minimal level of prior knowledge reducing the Bias and RMSE in point estimation and achieving near 95% coverage while shortening the length in interval estimation.

We also examined how the uncertainty in the MMT would affect the RR estimation using the MMT as a reference temperature. We derived the empirical distribution of the MMT-referenced RR through a sampling procedure similar to deriving the MMT distribution. Then, the empirical mean and percentiles were used as alternative point and interval estimates for the RR with the uncertainty in the MMT accounted for. Compared with the current approach (using only a single point estimate for the MMT as a reference value in quantifying the RR and calculating the confidence interval based on the normal approximation), the empirical RR estimates, when prior knowledge is combined, were less biased with reduced RMSE and achieves appropriate level of coverage probability in most of the scenarios.

Our proposed approach conceptually relies on a Bayesian inferential framework but is not a fully Bayesian hierarchical model, which one may consider as a more natural way to incorporate a prior knowledge in the inference for MMT. However, our approach has several advantages compared with constructing a fully Bayesian model. When modeling a nonlinear association between temperature and mortality using splines, MMT is not a specific parameter but a complex function of parameters (i.e., $\underset{x}{\mathrm{argmin}\Big(}{\boldsymbol{Q}}_x\boldsymbol{\beta} \Big)$ where ***β*** is reduced coefficients from ***η***, which is the original coefficients for the cross-basis, and ***Q***_*x*_ is the vector of basis variables as in formula (3)) and, thus a prior cannot be directly assigned on the MMT in a fully Bayesian model. Although indirect specification through a prior on ***η*** may be possible, a common choice of prior on ***η*** (e.g., multivariate normal) does not yield a known or closed form of prior on the MMT, which makes it difficult to incorporate prior knowledge on the MMT straightforwardly. In addition, in a time-series analysis for the temperature-mortality association, there are many other terms in the model to adjust for long-term trend, seasonality, and potential confounders. A fully Bayesian model would require a prior assumption and a posterior sampling on the whole parameter space including these parameters, which is often high-dimensional. In contrast, our approach focuses only on the cross-basis terms for temperature, which does not only facilitate the procedure of inserting a prior knowledge on the MMT but also avoids such an unnecessary high-dimensional posterior sampling for the nuisance parameters.

While our proposed approach has several benefits, some limitations should be acknowledged. First, if prior distribution is incorrectly specified, the whole inference can seriously be biased. To avoid such prior misspecification, one may use a prior information minimally by setting a potential range as the 1st – 99th percentiles. Our simulation showed that even with the minimally informative prior, the proposed method tends to perform better than the previous approach. When analyzing the US data, adding such minimal prior information reduced the uncertainty in the MMT by large amount particularly when the estimated association curves are unstable in terms of suggesting an MMT (e.g., category 4). Second, our approach limits the prior choice to be a Uniform distribution with different supports reflecting different prior knowledge. Such prior cannot accommodate a case where a prior range is the whole temperature range with different prior probabilities across the range, which would be more plausible in practice. Although a Uniform with a truncated support would be a reasonable approximation, further research is merited to improve the method to encompass a broader range of prior knowledge.

Applying the methods to the US data, we found four categories in terms of the MMT uncertainty. For categories 1 and 2, the estimated temperature-mortality association is mostly U-shape with short or long arms on either side and with a short bottom. In these categories, the MMT uncertainty is small and estimated between the 75th through the 95th percentiles of the observed temperature distribution. For category 3, the association is mostly reverse J-shape with relatively long bottom on the right side and the MMT uncertainty was relatively large. The large uncertainty is induced by the long bottom of the association curve and it may be more appropriate to describe the MMT as a range, not a single point. The previous study [6] also suggested to introduce this new concept of the minimum mortality temperature range. In category 4, the association is the rotated S-shape with the left arm curving down at the lowest temperature, for which the uncertainty is very large. Such uncertainty on the left arm is induced by the sparse data and causes the MMT uncertainty to unnecessarily cover the whole range of the temperature. In this case, it is suggested that adding restrictions on the range would lead to more reasonable inference on the MMT.

Finally, an important note should be made about the MMT estimation in a DLNM modeling framework. When the temperature-mortality association is incorrectly modeled (e.g., misspecifications of the splines and knots), either the previous or alternative approaches may provide interval estimates with significantly low coverage as they can miss the true MMT just below/above the lower/upper bounds. Since the interval estimation for the MMT can be sensitive to the modeling choices in DLNM depending on the temperature-mortality association pattern, one should carefully conduct a model selection procedure to identify the correct association and, thus the true MMT. Additionally, miss-specifying the outcome distribution such as a simple adjustment for overdispersion with Quasi-Poisson family may also affect the inference, and more flexible statistical methods may be considered to account for it in further research [15].

## Conclusions

In summary, Monte Carlo simulation-based approach to estimate the MMT either as a point or as an interval was shown to be a reasonable approach, particularly when some prior restrictions are added to reduce the uncertainty. The MMT uncertainty can affect the estimation for the MMT-referenced RR and ignoring the MMT uncertainty in the RR estimation may lead to invalid results with respect to bias in point estimation and the nominal coverage in interval estimation.

## Additional file

Additional file 1: Supplemental Materials. (PDF 3700 kb)

## Abbreviations

%CP: Coverage probability
DLNM: Distributed lag nonlinear model
MMT: Minimum mortality temperature
RMSE: Root mean squared error
RR: Relative risk
