# Supplementary material for: Monte Carlo simulation-based estimation for the minimum mortality temperature in temperature-mortality association study
Source: BMC Med Res Methodol. 2017 Sep 7;17:137. doi: 10.1186/s12874-017-0412-7 (PMC5590173; doi:10.1186/s12874-017-0412-7)
Supplement: Additional file 1: — Supplemental Materials. (PDF 3700 kb) [file 12874_2017_412_MOESM1_ESM.pdf]

Supplemental Materials

Monte Carlo simulation-based estimation for the minimum mortality temperature in temperature-mortality association study

Whanhee Lee, Ho Kim, Sunghee Hwang,

Antonella Zanobetti, Joel Schwartz, Yeonseung Chung

List of Contents

Figure S1. The true relative risk (RR) curves in four different scenarios considered in simulation study. Black vertical line indicates the true MMT. Red, green, and blue vertical lines indicate the prior ranges for the MMT incorporated in $\text{Empirical2}_{\text{strong}}$, $\text{Empirical2}_{\text{moderate}}$, and $\text{Empirical2}_{\text{minimal}}$.

Figure S2. Root mean squared error (RMSE) in estimating the minimum mortality temperature (MMT) by different methods for different sample sizes in each scenario.

Figure S3. The coverage probability (%CP) in estimating the minimum mortality temperature (MMT) by different methods for different sample sizes in each scenario.

Figure S4. Root mean squared error (RMSE) in estimating the minimum mortality temperature (MMT) by different methods for different specifications of the splines in each scenario.

Figure S5. The coverage probability (%CP) in estimating the minimum mortality temperature (MMT) by different methods for different specifications of the splines in each scenario.

Figure S6. Root mean squared error (RMSE) in estimating the minimum mortality temperature (MMT) by different methods for different specifications of the knots in quadratic B-spline in each scenario.

Figure S7. The coverage probability (%CP) in estimating the minimum mortality temperature

(MMT) by different methods for different specifications of the knots in quadratic B-spline in each scenario.

Figure S8. City-specific relative risk (RR) curve with the MMT estimated by different methods. Red cross indicates the point estimate of MMT calculated by Argmin2. Blue and orange vertical lines indicate the point and interval estimates calculated by Empirical1 and Empriciral2$_{minimal}$.

Table S1. Descriptive Statistics for mortality and temperature in the 135 cities of the United States.

Figure S1. The true relative risk (RR) curves in four different scenarios considered in simulation study. Black vertical line indicates the true MMT. Red, green, and blue vertical lines indicate the prior ranges for the MMT incorporated in Empirical2$_{\text{strong}}$, Empirical2$_{\text{moderate}}$, and Empirical2$_{\text{minimal}}$.

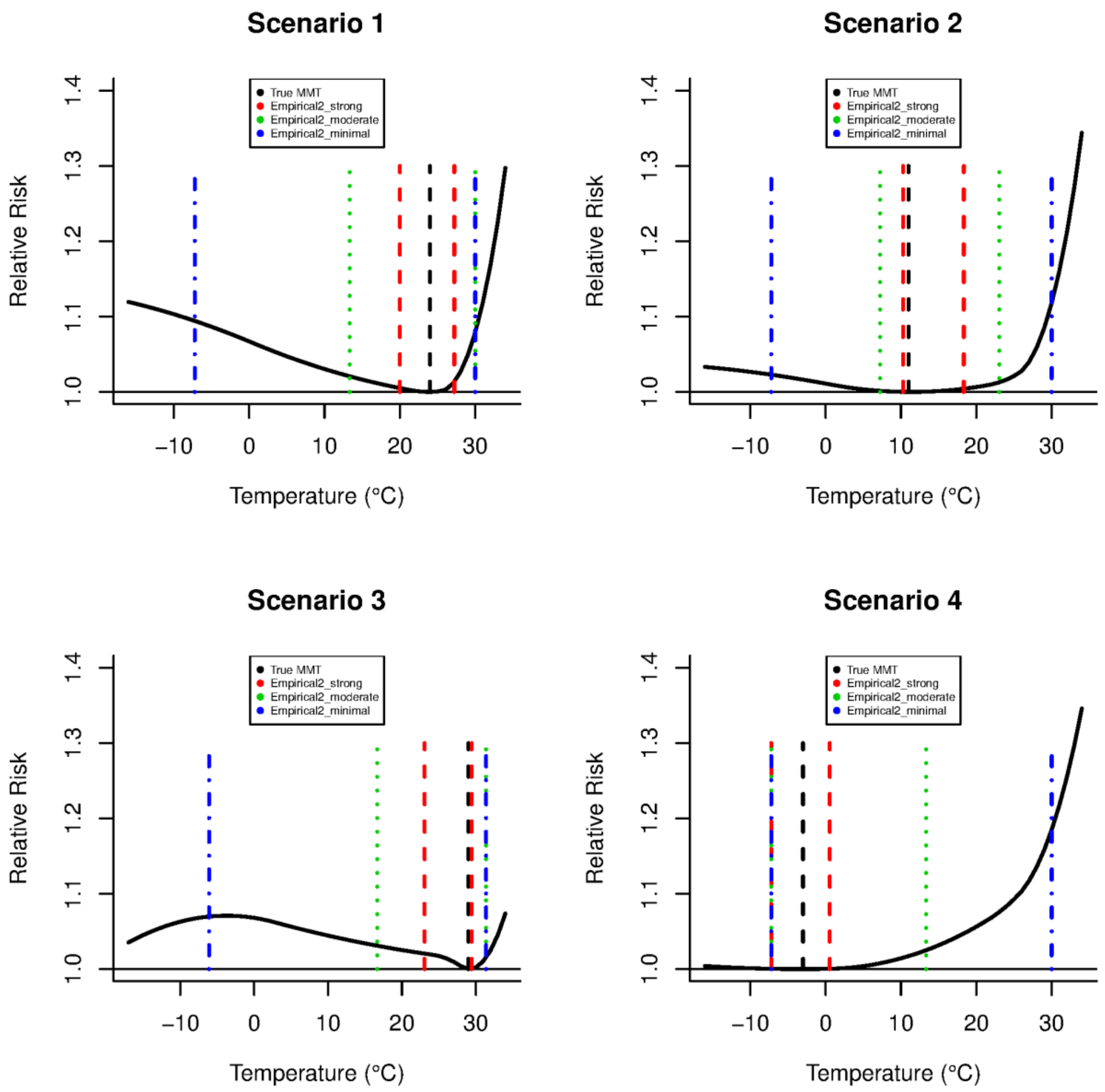

Figure S2. Root mean squared error (RMSE) in estimating the minimum mortality temperature (MMT) by different methods for different sample sizes in each scenario.

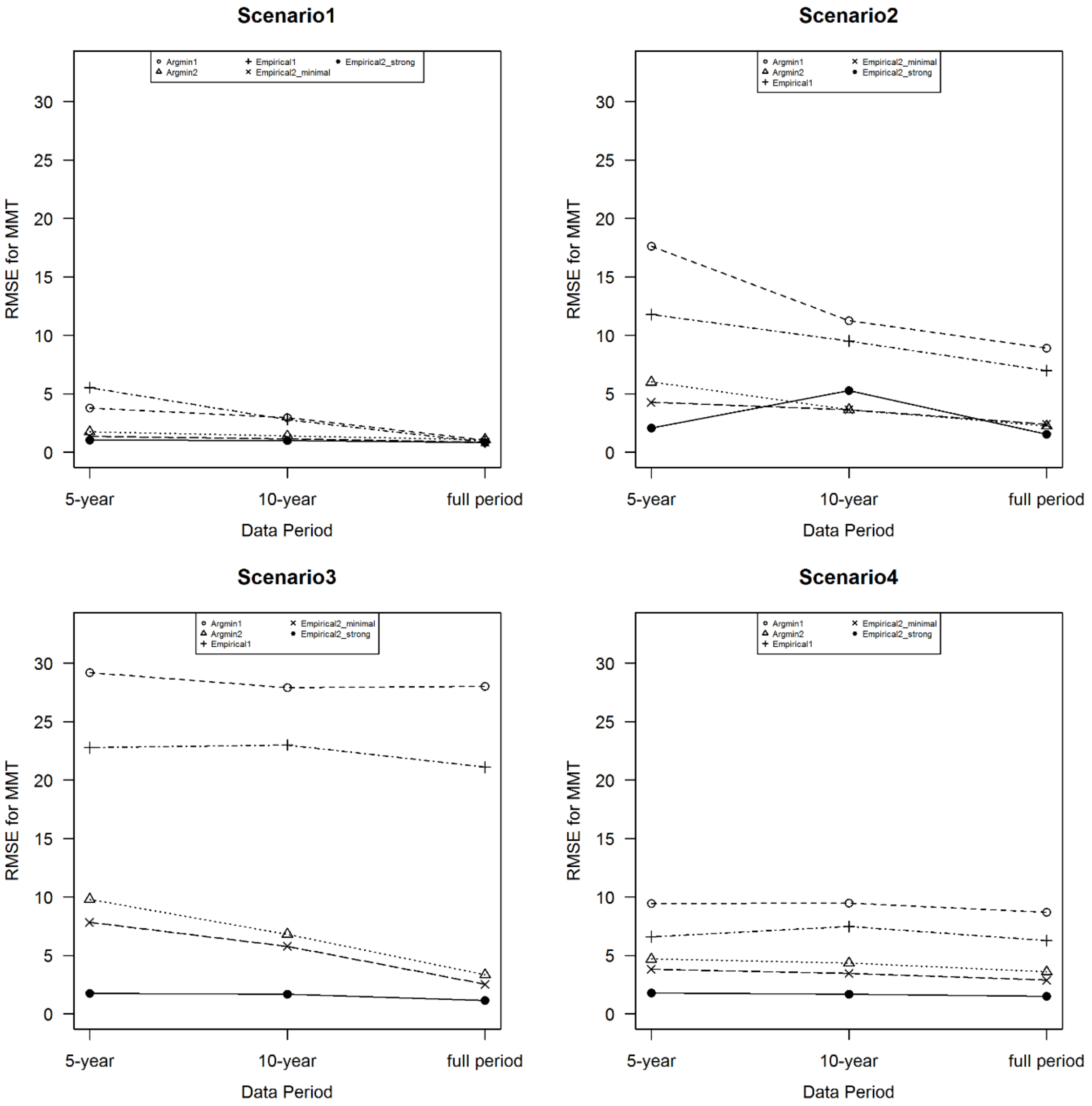

Figure S3. The coverage probability (%CP) in estimating the minimum mortality temperature (MMT) by different methods for different sample sizes in each scenario.

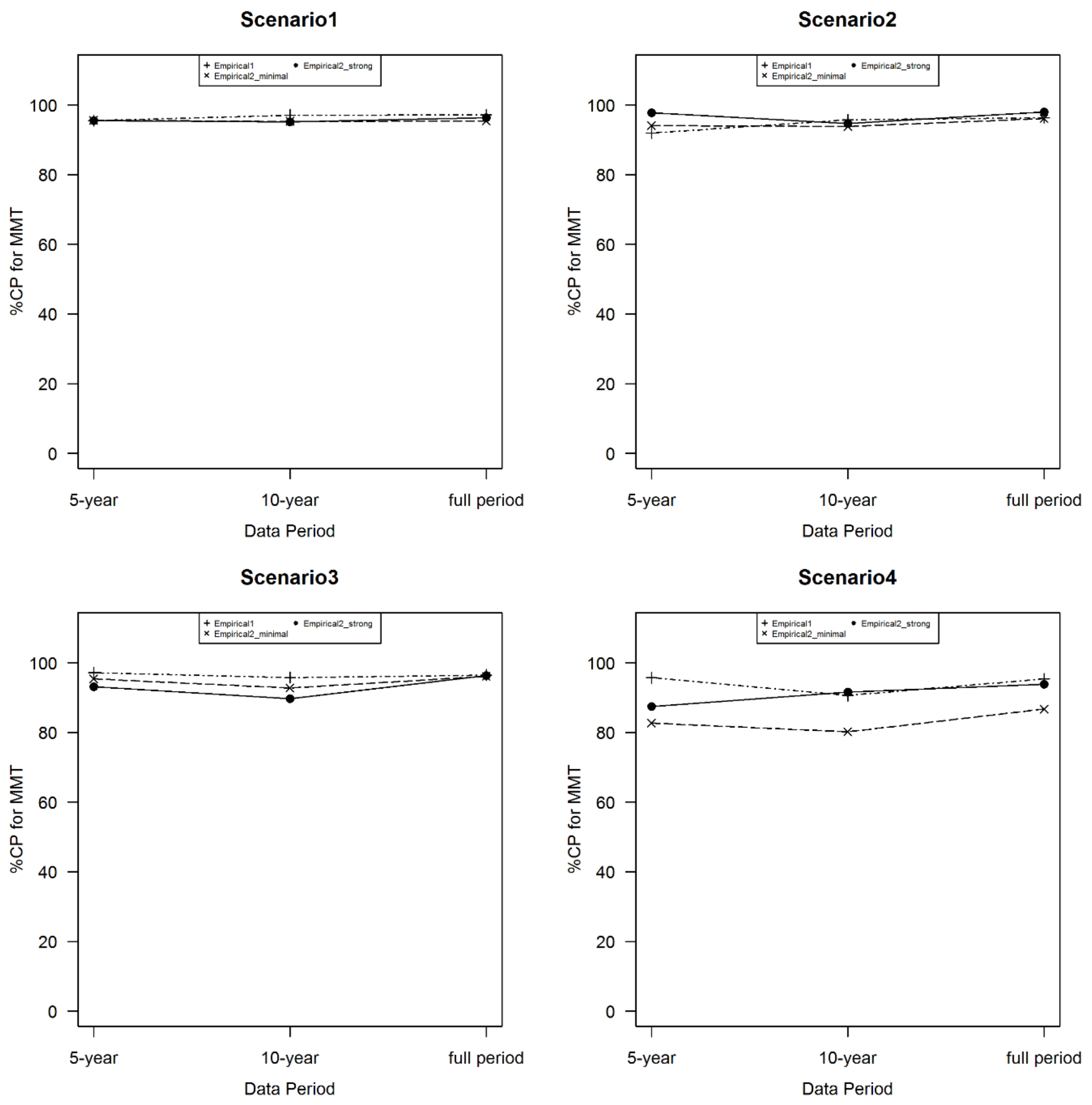

Figure S4. Root mean squared error (RMSE) in estimating the minimum mortality temperature (MMT) by different methods for different specifications of the splines in each scenario.

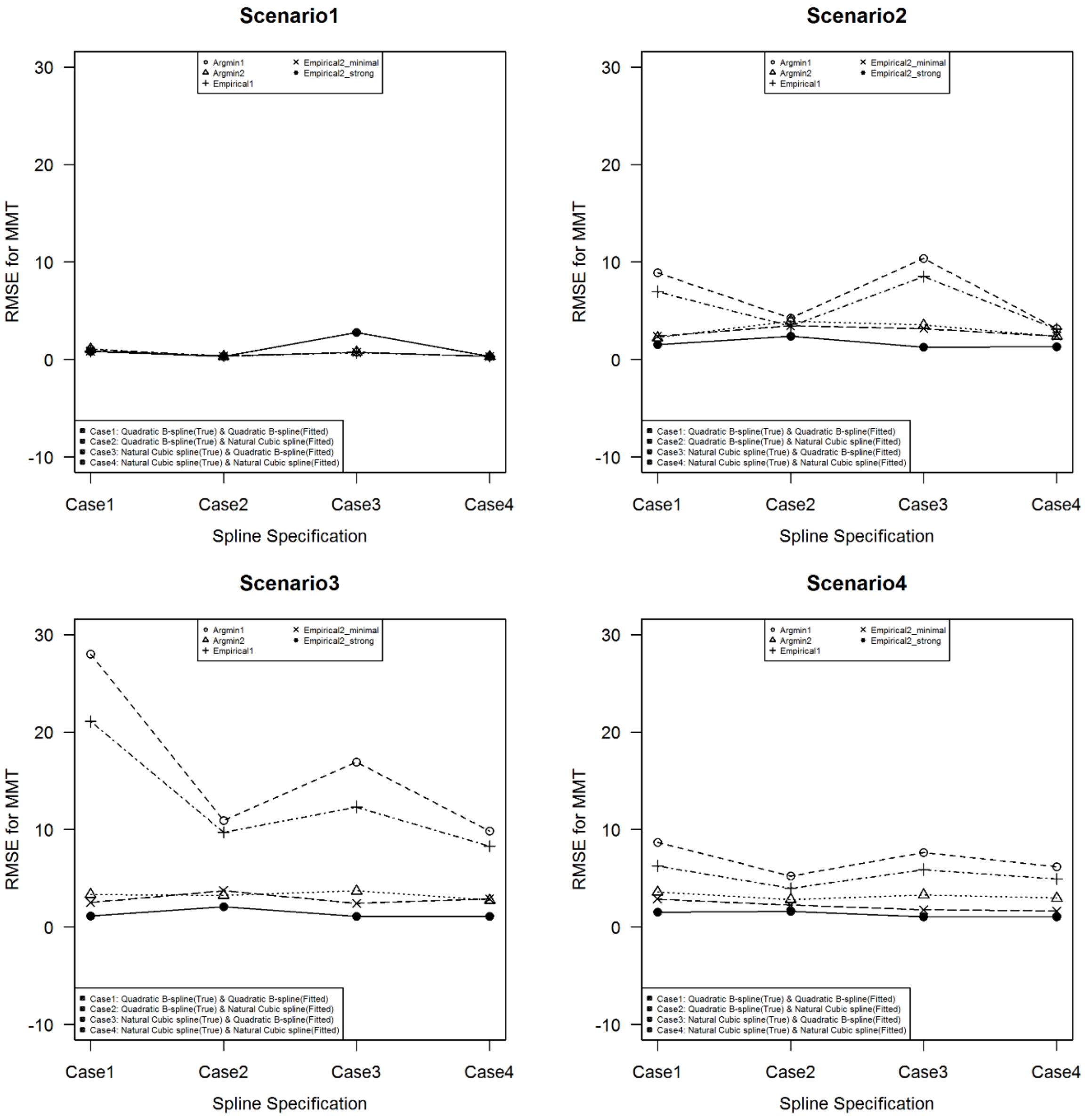

Figure S5. The coverage probability (%CP) in estimating the minimum mortality temperature (MMT) by different methods for different specifications of the splines in each scenario.

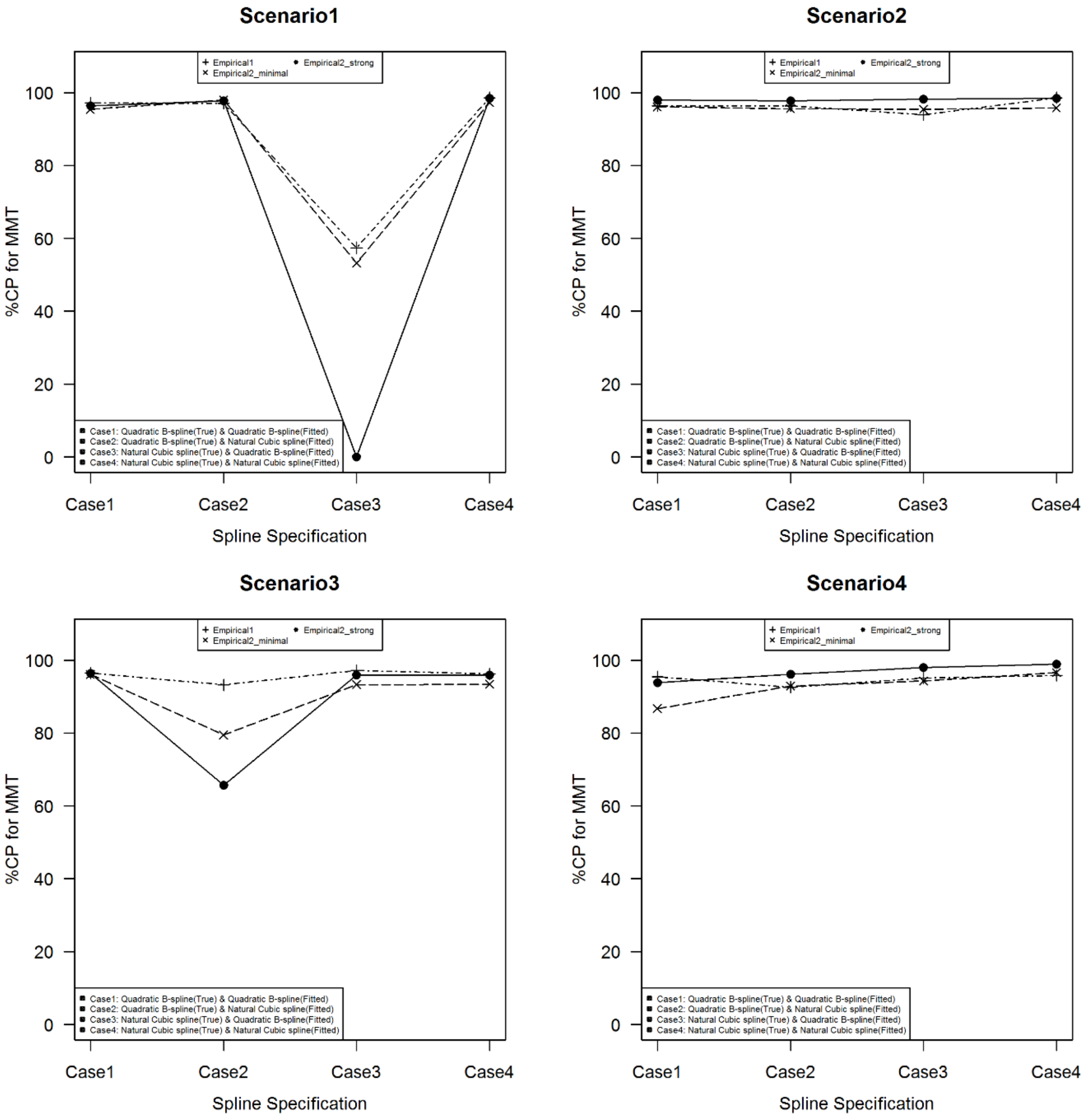

Figure S6. Root mean squared error (RMSE) in estimating the minimum mortality temperature (MMT) by different methods for different specifications of the knots in quadratic B-spline in each scenario.

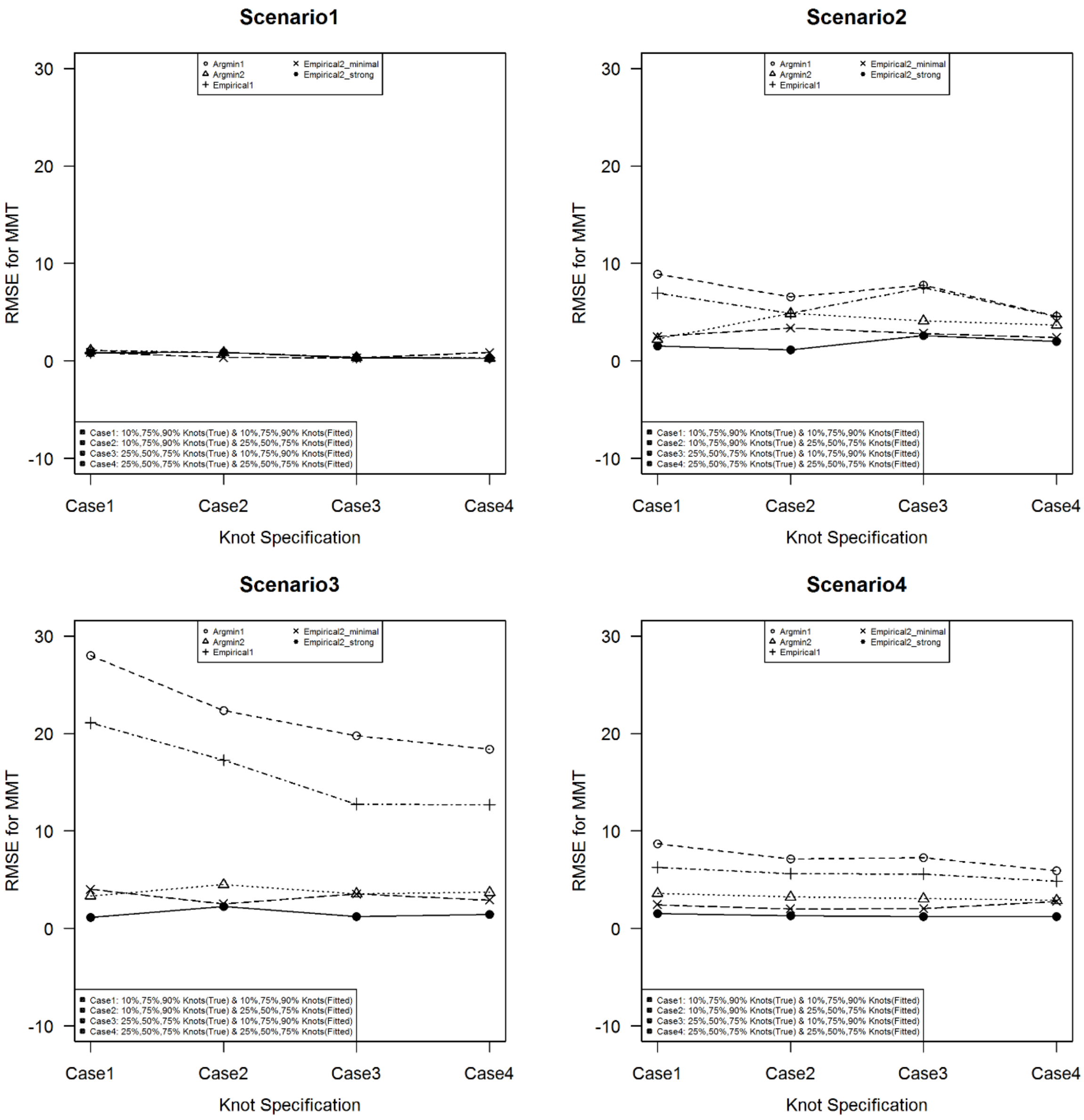

Figure S7. The coverage probability (%CP) in estimating the minimum mortality temperature (MMT) by different methods for different specifications of the knots in quadratic B-spline in each scenario.

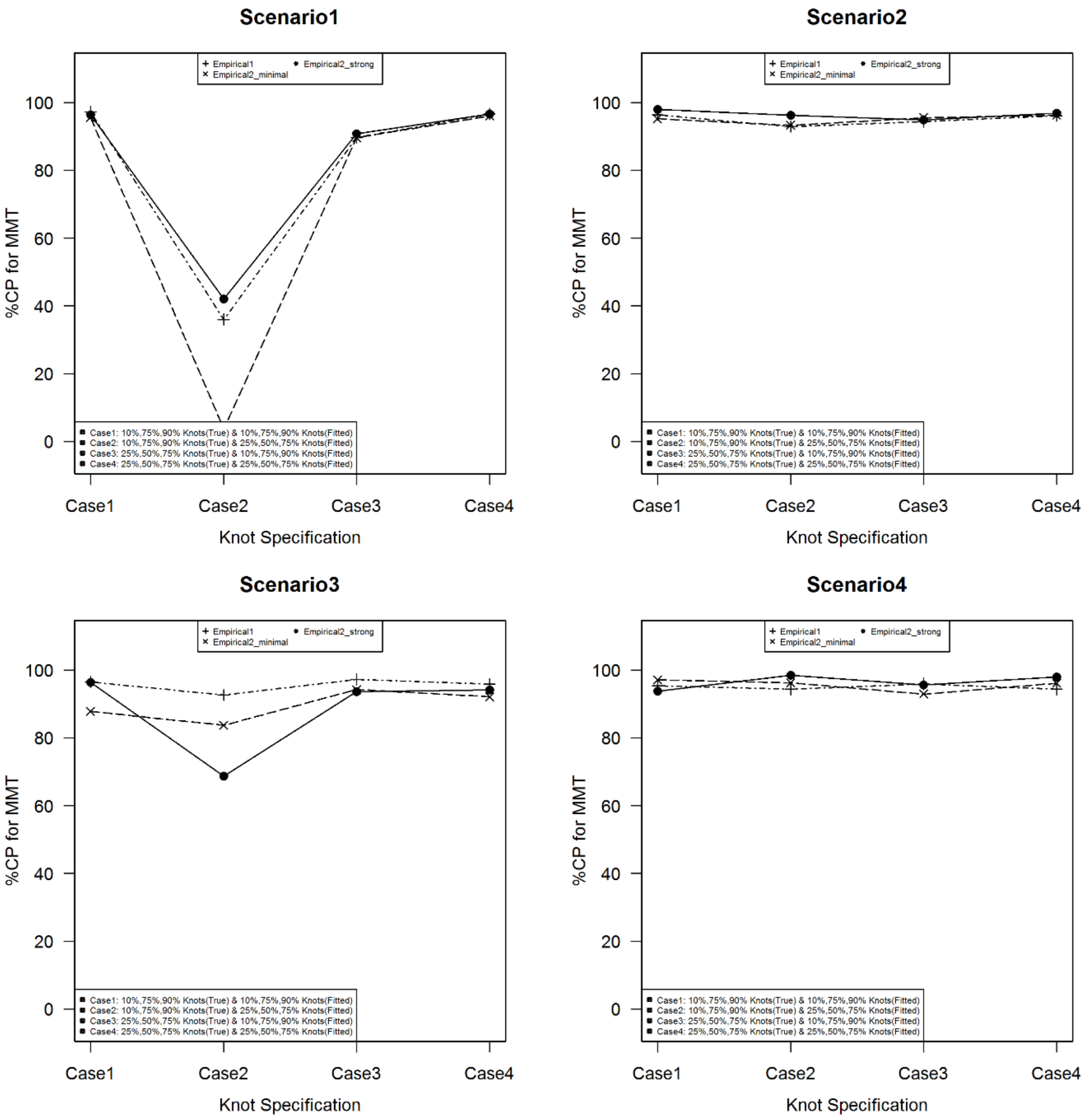

Figure S8. City-specific relative risk (RR) curve with the MMT estimated by different methods. Red cross indicates the point estimate of MMT calculated by Argmin2. Blue and orange vertical lines indicate the point and interval estimates calculated by Empirical1 and Empricir al2$_{minimal}$.

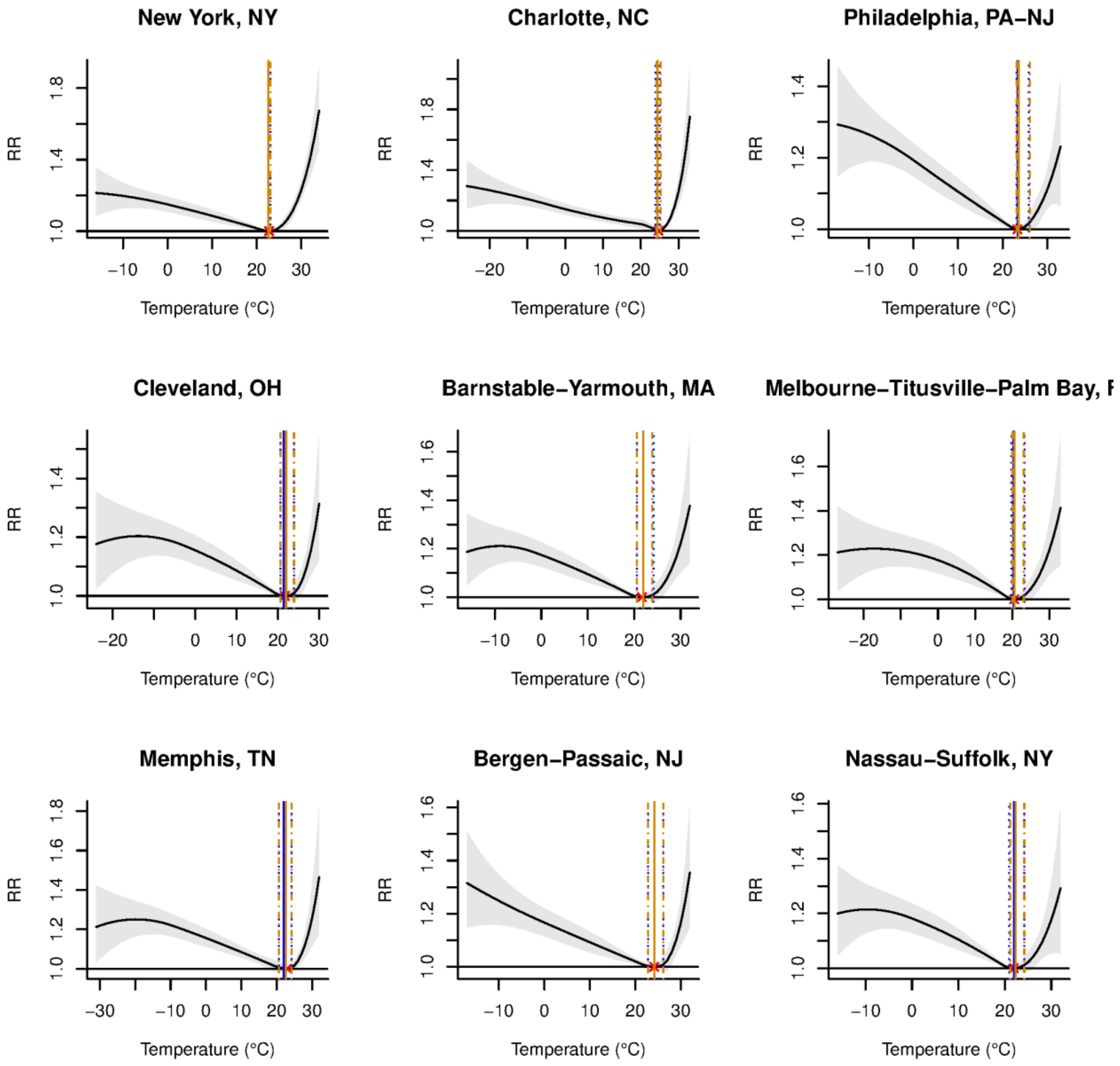

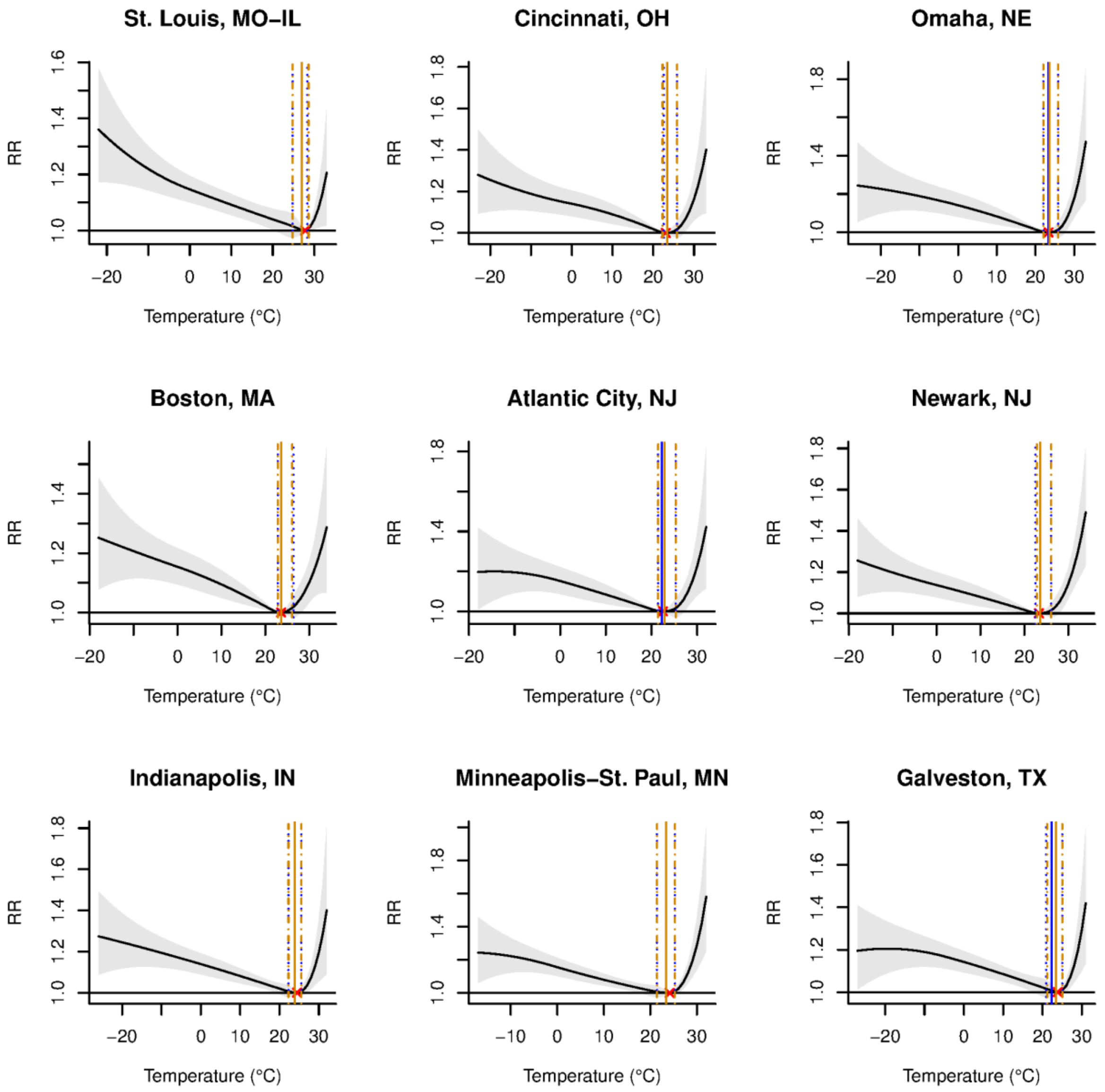
St. Louis, MO–IL
Cincinnati, OH
Omaha, NE
Boston, MA
Atlantic City, NJ
Newark, NJ
Indianapolis, IN
Minneapolis–St. Paul, MN
Galveston, TX
RR
Temperature (°C)
−20
−10
0
10
20
30
1.0
1.2
1.4
1.6
1.8

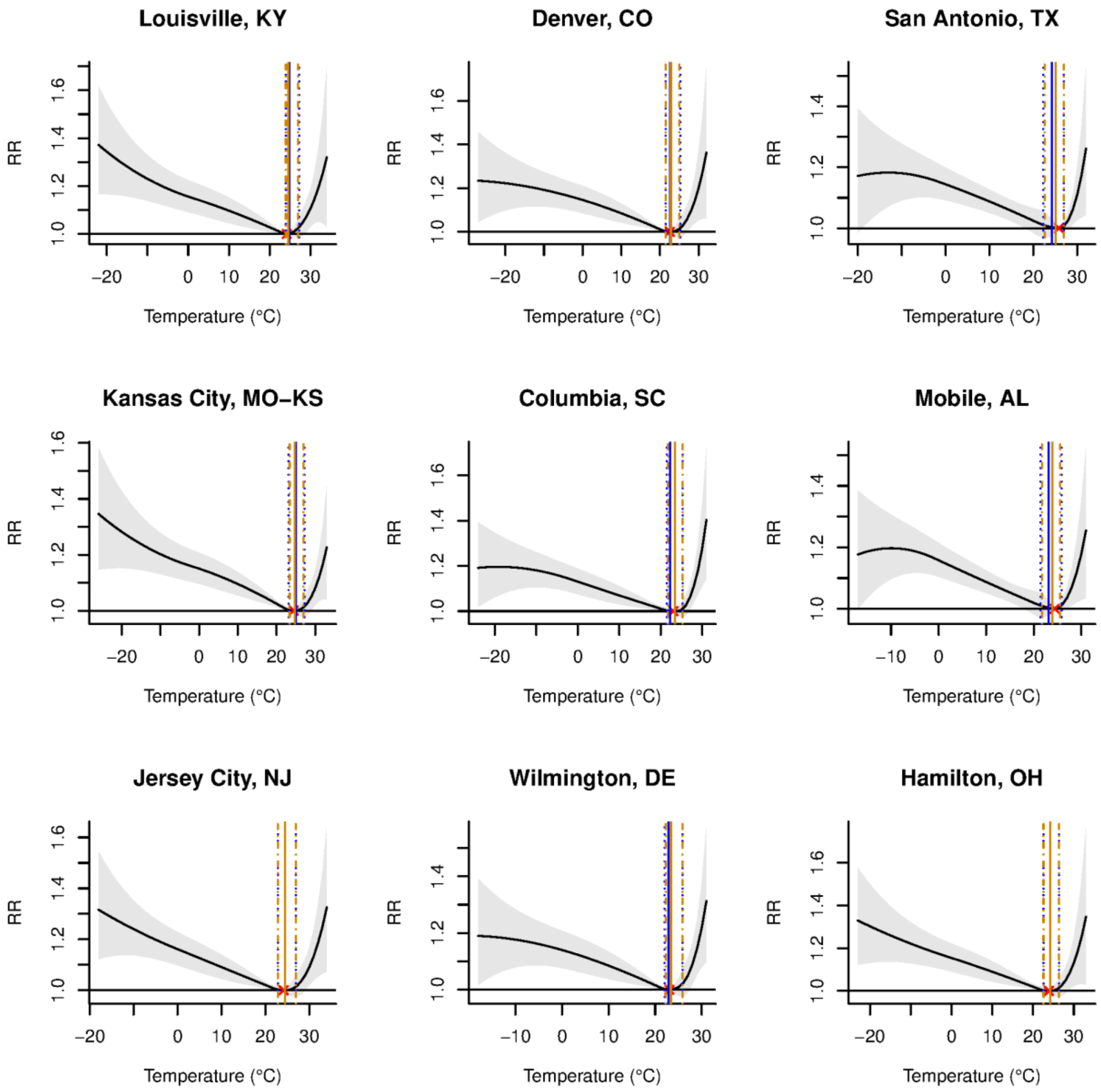

Louisville, KY
Denver, CO
San Antonio, TX
Kansas City, MO–KS
Columbia, SC
Mobile, AL
Jersey City, NJ
Wilmington, DE
Hamilton, OH
RR
Temperature (°C)

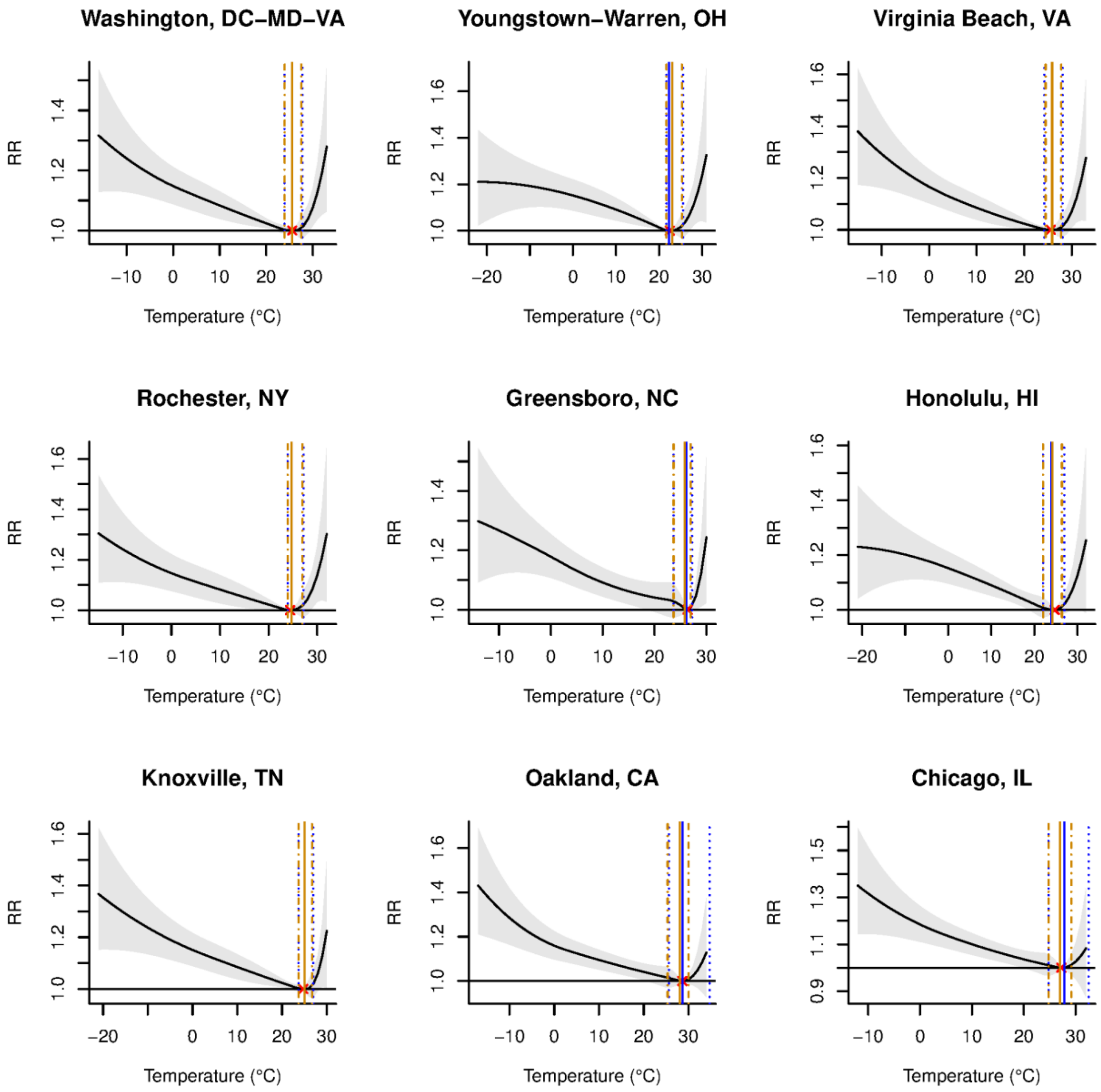
Washington, DC−MD−VA
Youngstown−Warren, OH
Virginia Beach, VA
Rochester, NY
Greensboro, NC
Honolulu, HI
Knoxville, TN
Oakland, CA
Chicago, IL
RR
Temperature (°C)

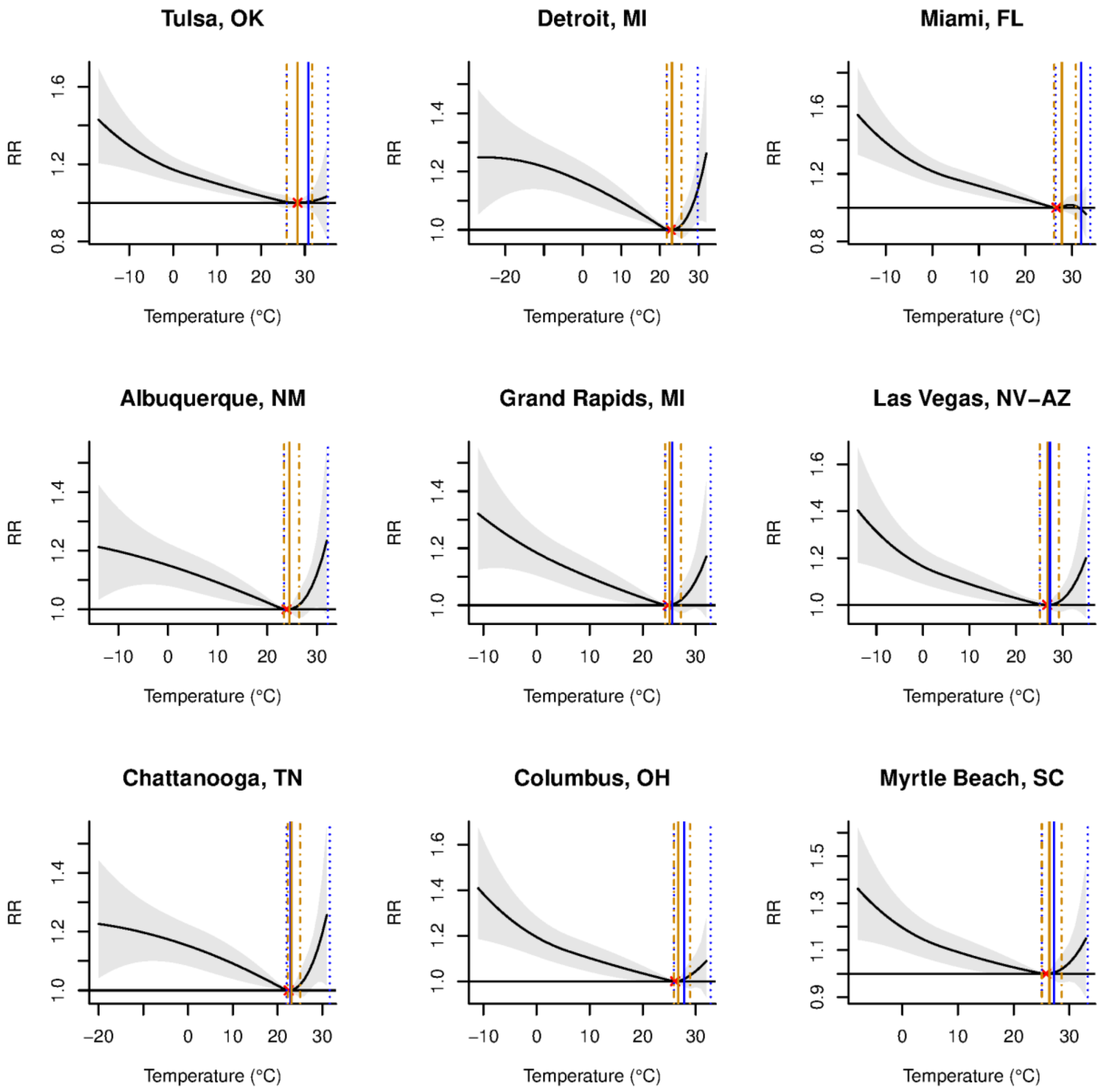
Tulsa, OK
RR
0.8
1.2
1.6
−10
0
10
20
30
Temperature (°C)
Detroit, MI
RR
1.0
1.2
1.4
−20
0
10
20
30
Temperature (°C)
Miami, FL
RR
0.8
1.2
1.6
−10
0
10
20
30
Temperature (°C)
Albuquerque, NM
RR
1.0
1.2
1.4
−10
0
10
20
30
Temperature (°C)
Grand Rapids, MI
RR
1.0
1.2
1.4
−10
0
10
20
30
Temperature (°C)
Las Vegas, NV−AZ
RR
1.0
1.2
1.4
1.6
−10
0
10
20
30
Temperature (°C)
Chattanooga, TN
RR
1.0
1.2
1.4
−20
0
10
20
30
Temperature (°C)
Columbus, OH
RR
1.0
1.2
1.4
1.6
−10
0
10
20
30
Temperature (°C)
Myrtle Beach, SC
RR
0.9
1.1
1.3
1.5
0
10
20
30
Temperature (°C)

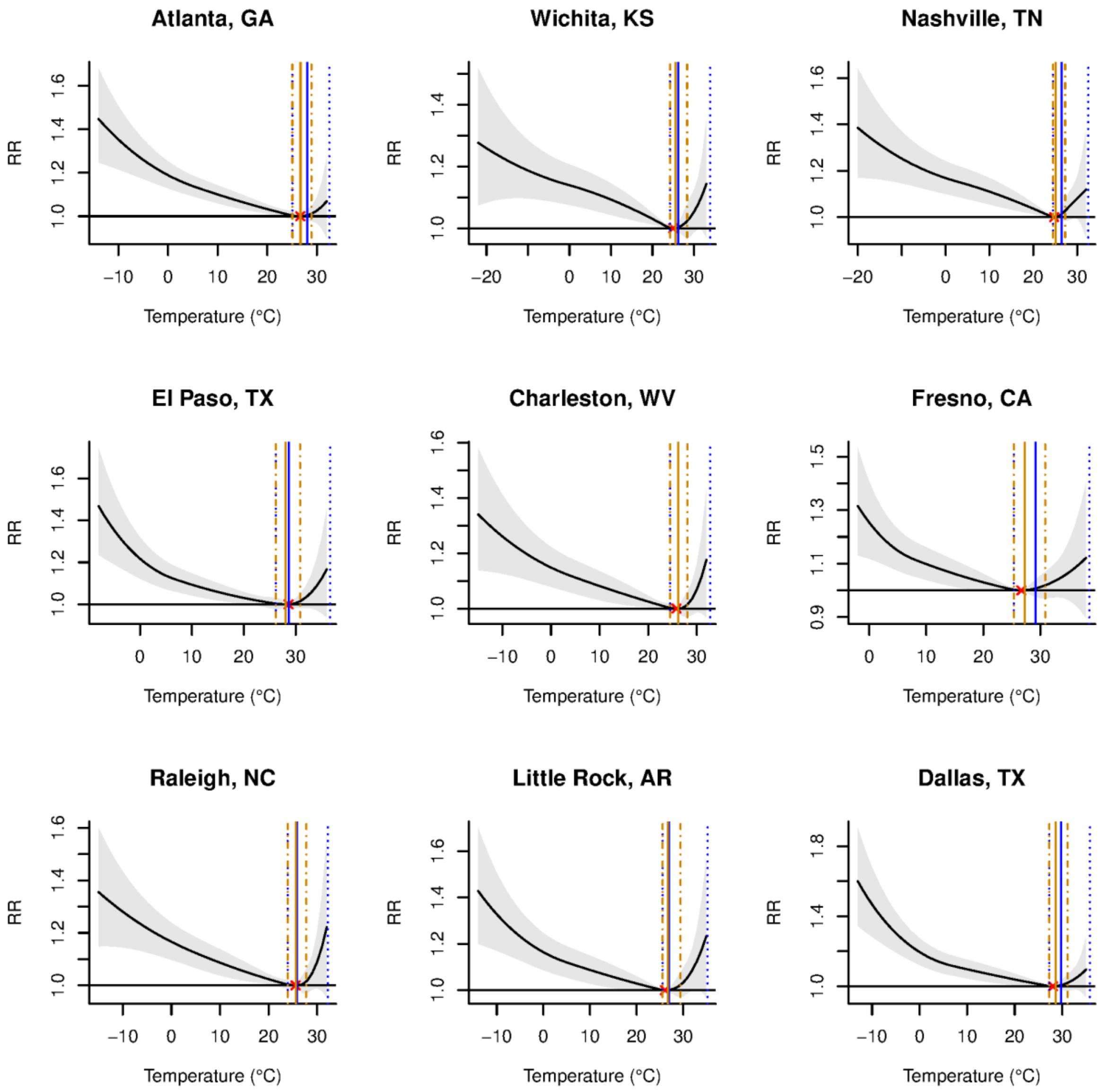
Atlanta, GA
Wichita, KS
Nashville, TN
El Paso, TX
Charleston, WV
Fresno, CA
Raleigh, NC
Little Rock, AR
Dallas, TX
RR
Temperature (°C)

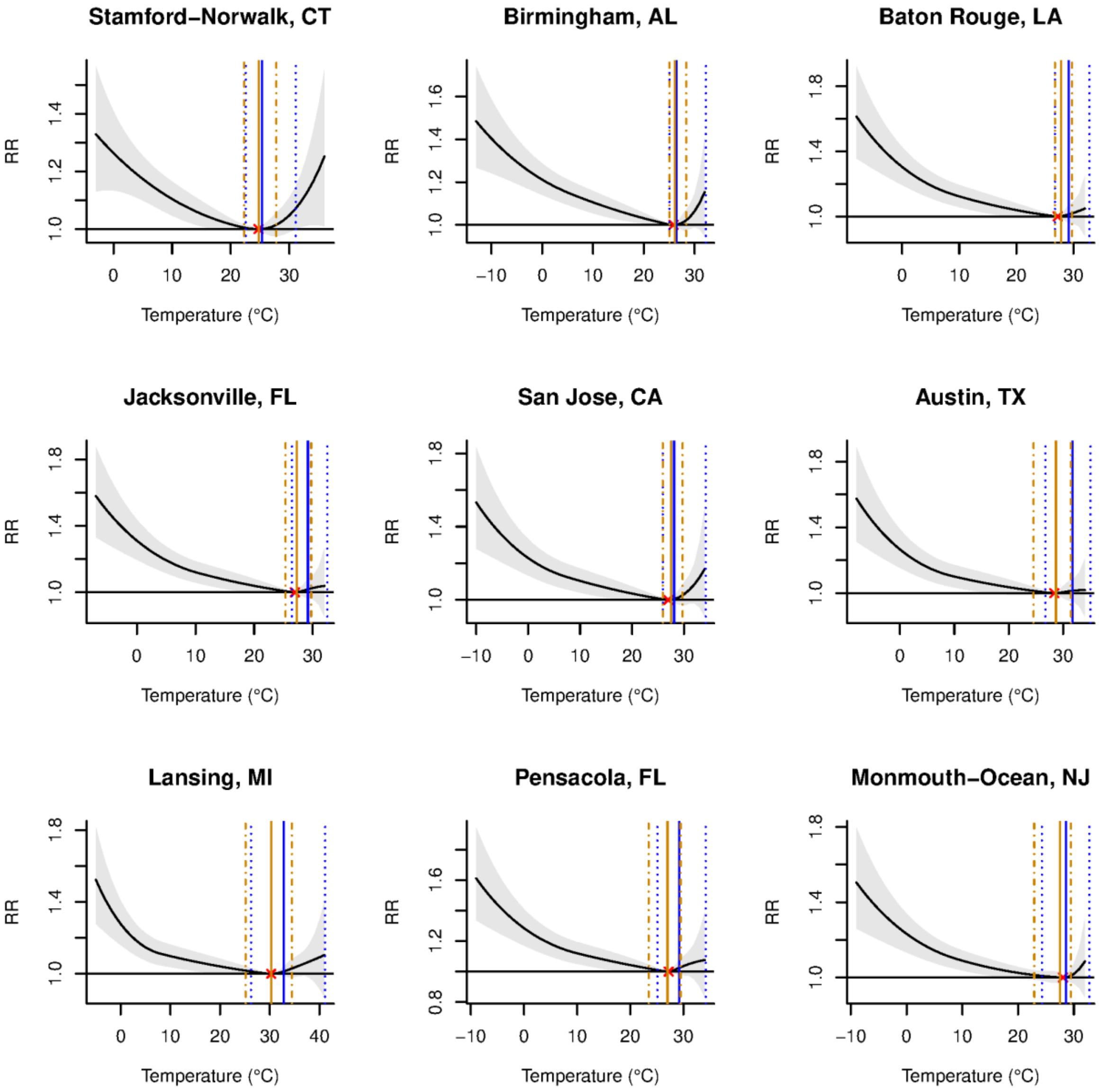
Stamford−Norwalk, CT
Birmingham, AL
Baton Rouge, LA
Jacksonville, FL
San Jose, CA
Austin, TX
Lansing, MI
Pensacola, FL
Monmouth−Ocean, NJ
RR
Temperature (°C)

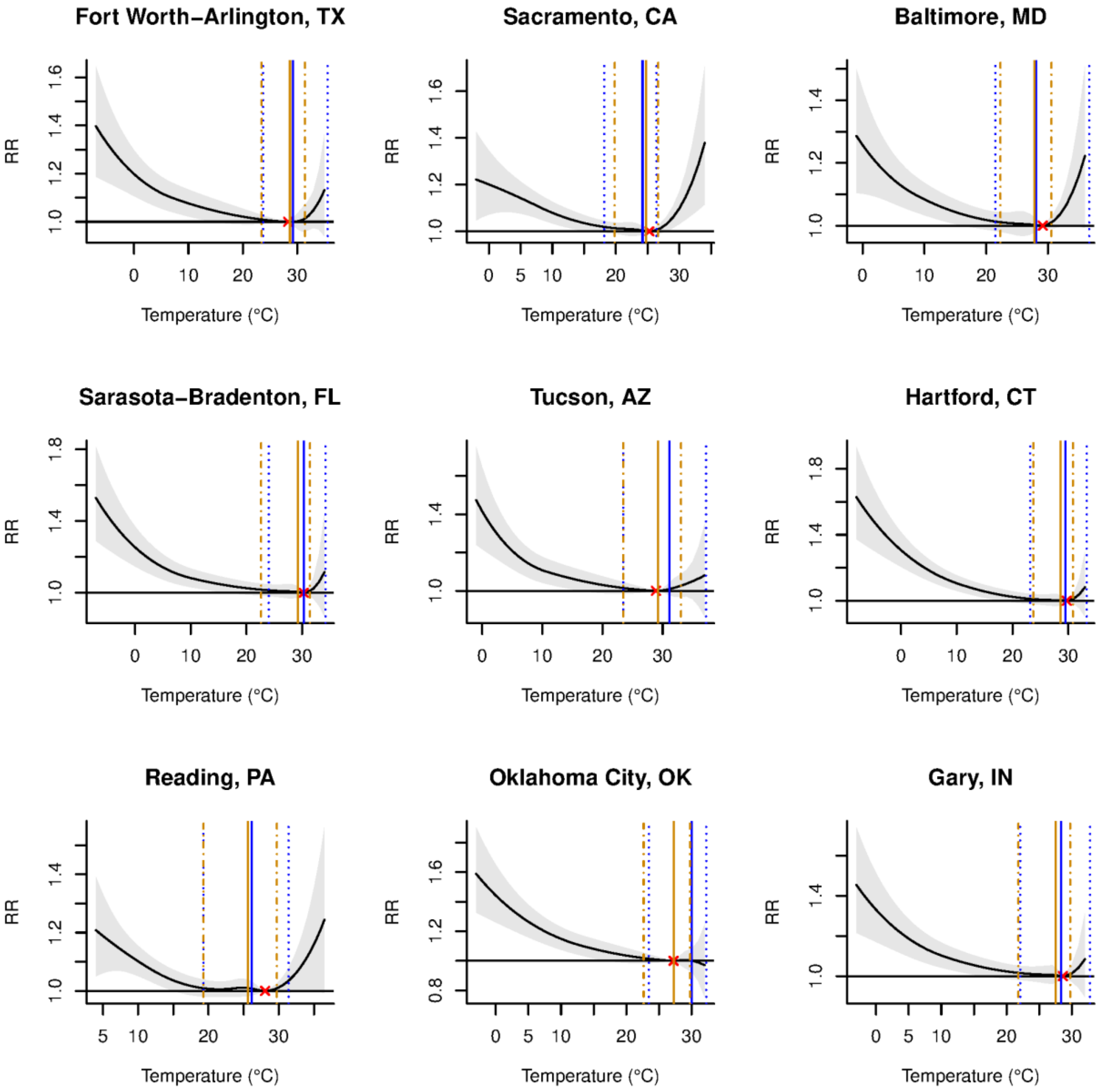

Fort Worth−Arlington, TX
Sacramento, CA
Baltimore, MD
Sarasota−Bradenton, FL
Tucson, AZ
Hartford, CT
Reading, PA
Oklahoma City, OK
Gary, IN
RR
Temperature (°C)

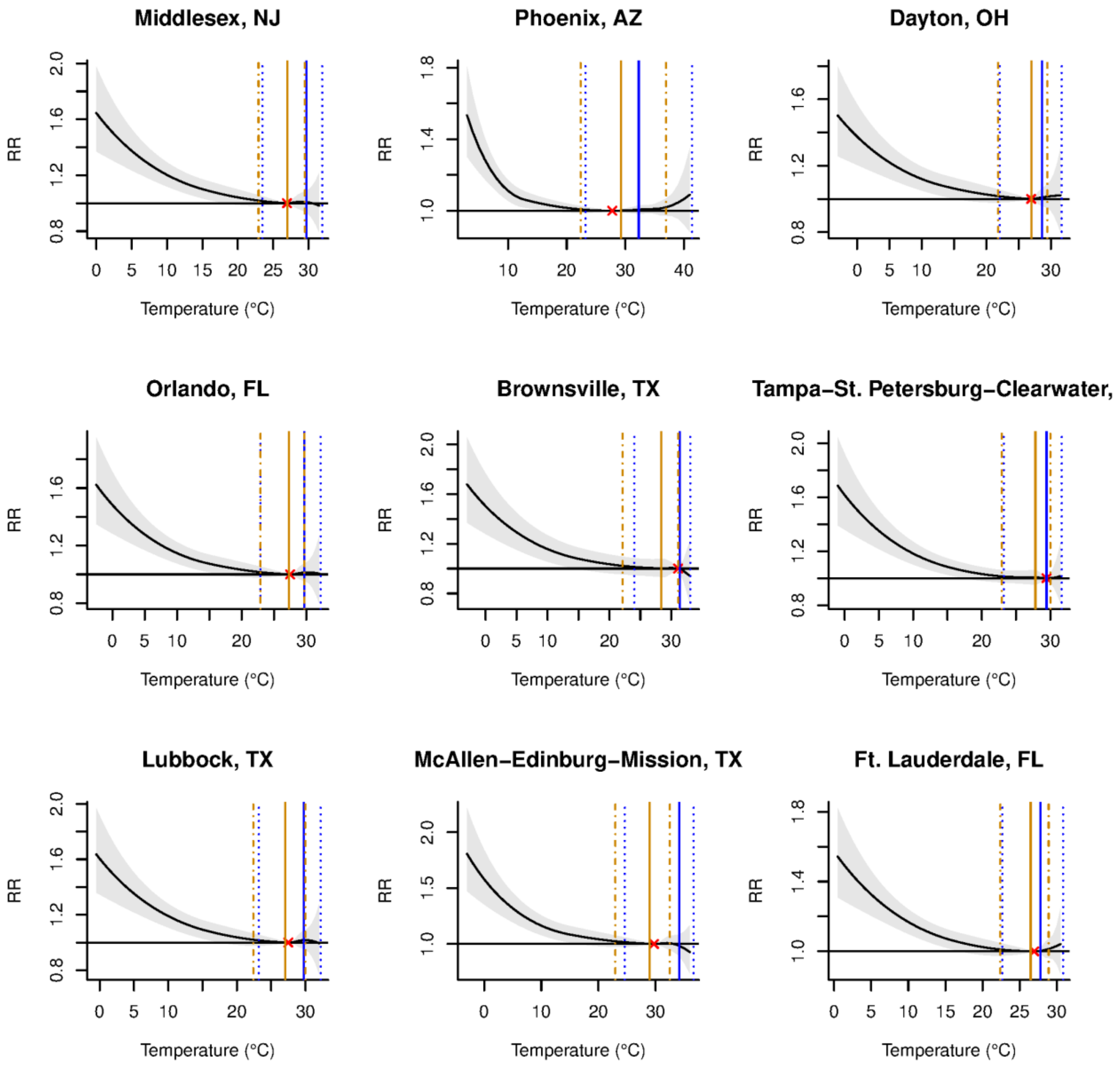

Middlesex, NJ
Phoenix, AZ
Dayton, OH
Orlando, FL
Brownsville, TX
Tampa−St. Petersburg−Clearwater,
Lubbock, TX
McAllen−Edinburg−Mission, TX
Ft. Lauderdale, FL
RR
Temperature (°C)

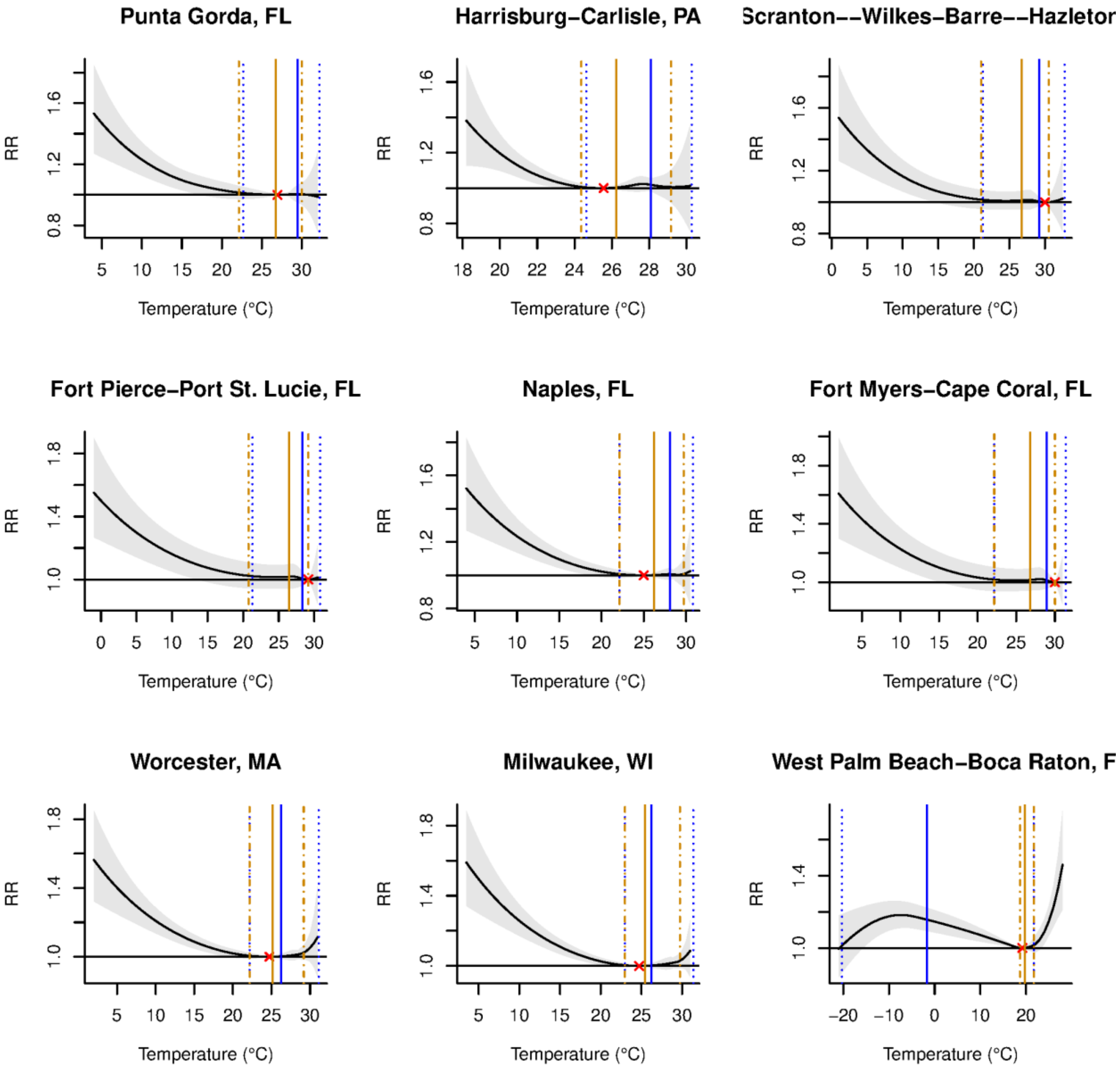

Punta Gorda, FL
Harrisburg–Carlisle, PA
Scranton––Wilkes–Barre––Hazleton
Fort Pierce–Port St. Lucie, FL
Naples, FL
Fort Myers–Cape Coral, FL
Worcester, MA
Milwaukee, WI
West Palm Beach–Boca Raton, F
RR
Temperature (°C)

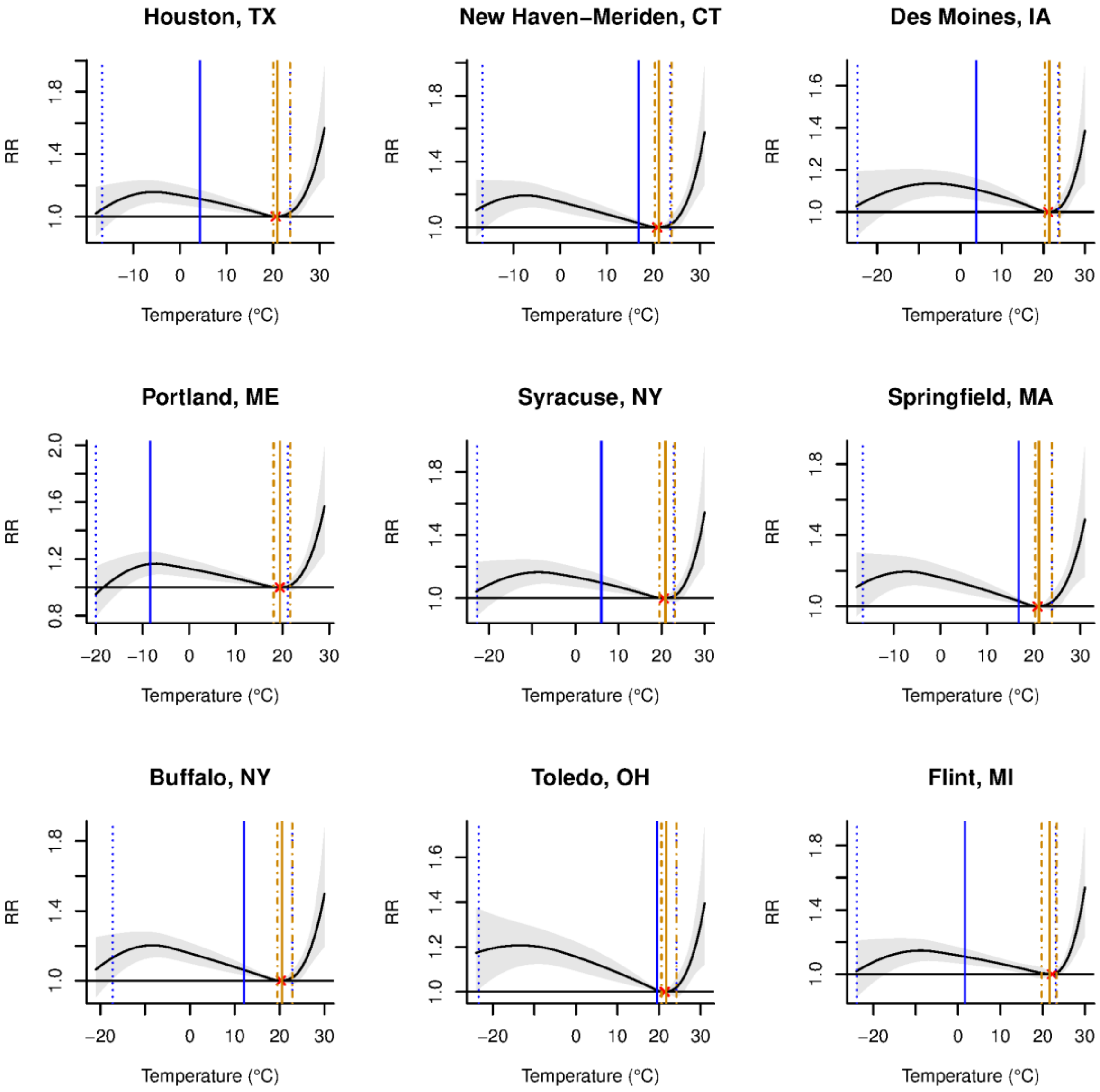

Houston, TX
RR
1.0 1.4 1.8
−10 0 10 20 30
Temperature (°C)
New Haven−Meriden, CT
RR
1.0 1.4 1.8
−10 0 10 20 30
Temperature (°C)
Des Moines, IA
RR
1.0 1.2 1.4 1.6
−20 0 10 20 30
Temperature (°C)
Portland, ME
RR
0.8 1.2 1.6 2.0
−20 −10 0 10 20 30
Temperature (°C)
Syracuse, NY
RR
1.0 1.4 1.8
−20 0 10 20 30
Temperature (°C)
Springfield, MA
RR
1.0 1.4 1.8
−10 0 10 20 30
Temperature (°C)
Buffalo, NY
RR
1.0 1.4 1.8
−20 0 10 20 30
Temperature (°C)
Toledo, OH
RR
1.0 1.2 1.4 1.6
−20 0 10 20 30
Temperature (°C)
Flint, MI
RR
1.0 1.4 1.8
−20 0 10 20 30
Temperature (°C)

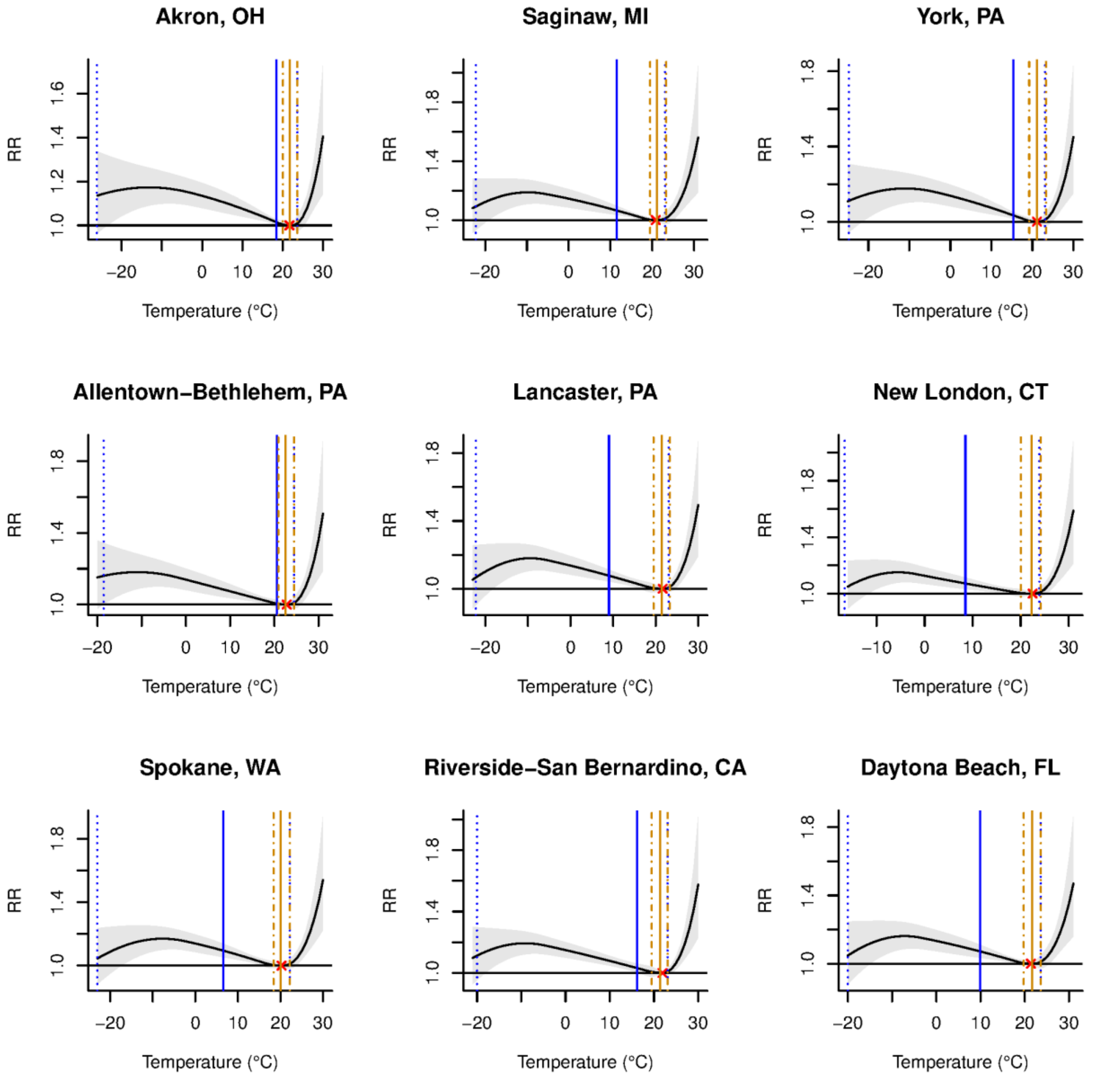

Akron, OH
RR
1.0 1.2 1.4 1.6
−20 0 10 20 30
Temperature (°C)
Saginaw, MI
RR
1.0 1.4 1.8
−20 0 10 20 30
Temperature (°C)
York, PA
RR
1.0 1.4 1.8
−20 0 10 20 30
Temperature (°C)
Allentown−Bethlehem, PA
RR
1.0 1.4 1.8
−20 0 10 20 30
Temperature (°C)
Lancaster, PA
RR
1.0 1.4 1.8
−20 0 10 20 30
Temperature (°C)
New London, CT
RR
1.0 1.4 1.8
−10 0 10 20 30
Temperature (°C)
Spokane, WA
RR
1.0 1.4 1.8
−20 0 10 20 30
Temperature (°C)
Riverside−San Bernardino, CA
RR
1.0 1.4 1.8
−20 0 10 20 30
Temperature (°C)
Daytona Beach, FL
RR
1.0 1.4 1.8
−20 0 10 20 30
Temperature (°C)

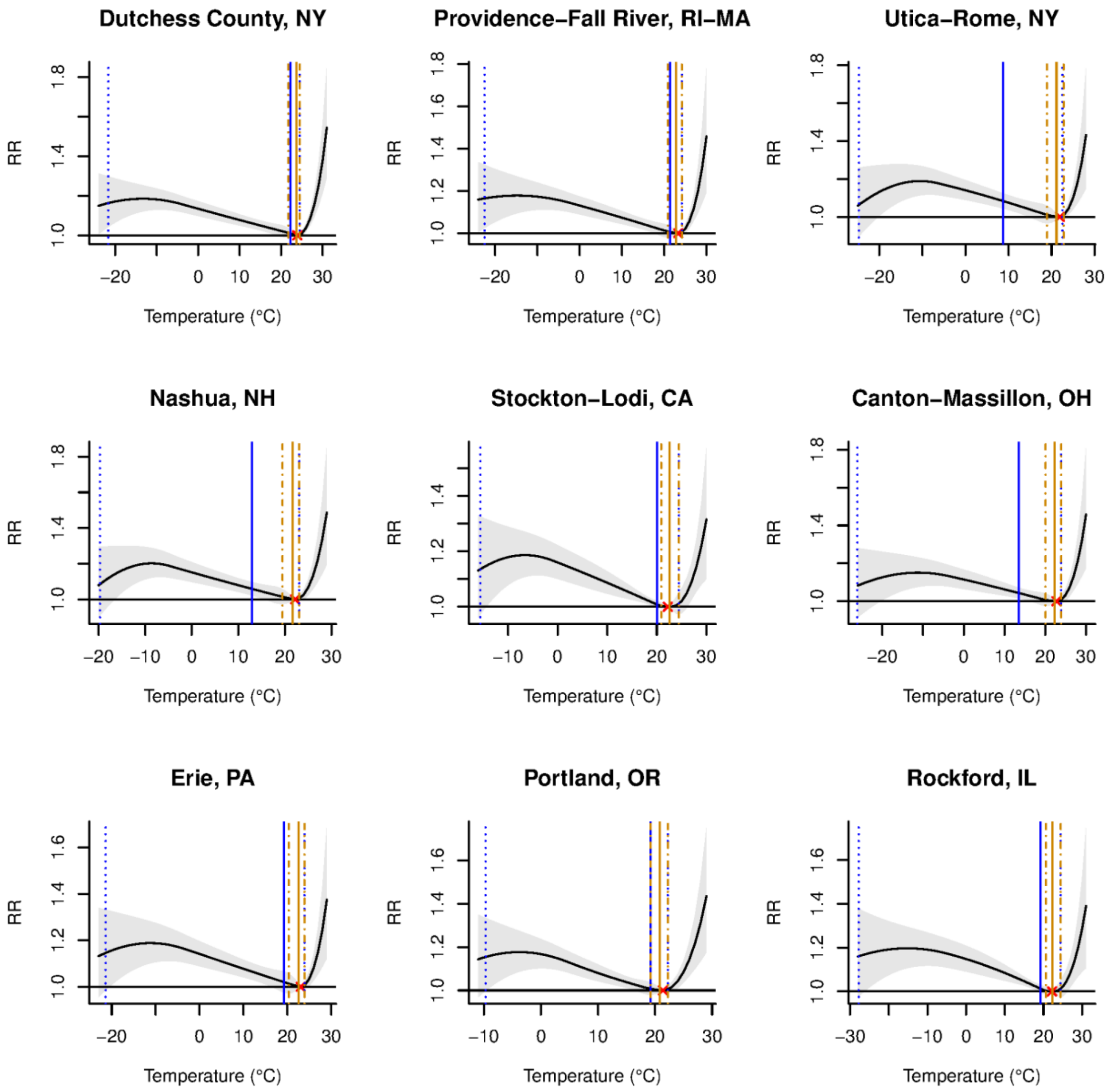
Dutchess County, NY
Providence−Fall River, RI−MA
Utica−Rome, NY
Nashua, NH
Stockton−Lodi, CA
Canton−Massillon, OH
Erie, PA
Portland, OR
Rockford, IL
RR
Temperature (°C)

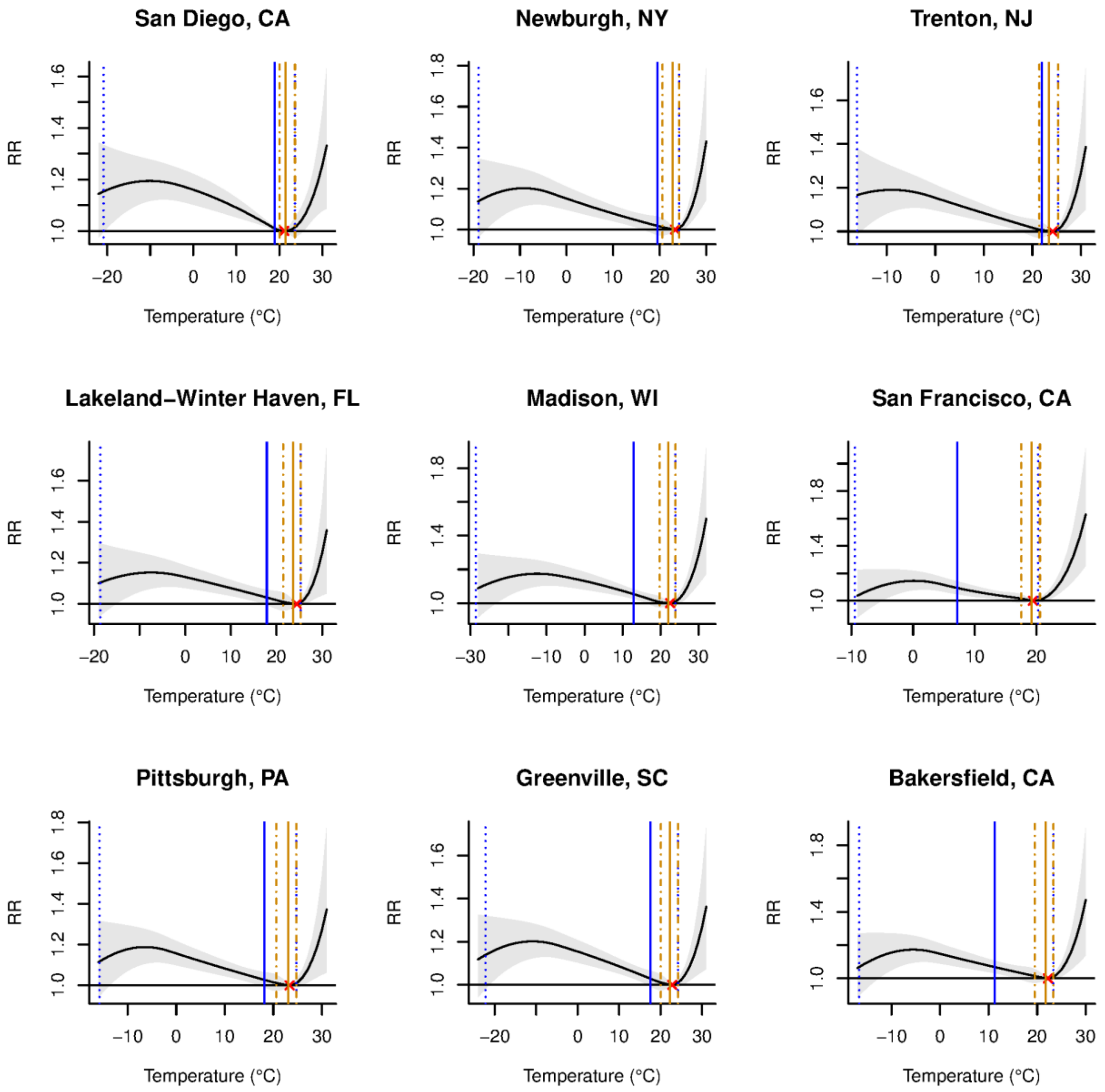

San Diego, CA
RR
1.0
1.2
1.4
1.6
−20
0
10
20
30
Temperature (°C)
Newburgh, NY
RR
1.0
1.2
1.4
1.6
1.8
−20
−10
0
10
20
30
Temperature (°C)
Trenton, NJ
RR
1.0
1.2
1.4
1.6
−10
0
10
20
30
Temperature (°C)
Lakeland−Winter Haven, FL
RR
1.0
1.2
1.4
1.6
−20
0
10
20
30
Temperature (°C)
Madison, WI
RR
1.0
1.4
1.8
−30
−10
0
10
20
30
Temperature (°C)
San Francisco, CA
RR
1.0
1.4
1.8
−10
0
10
20
Temperature (°C)
Pittsburgh, PA
RR
1.0
1.2
1.4
1.6
1.8
−10
0
10
20
30
Temperature (°C)
Greenville, SC
RR
1.0
1.2
1.4
1.6
−20
0
10
20
30
Temperature (°C)
Bakersfield, CA
RR
1.0
1.4
1.8
−10
0
10
20
30
Temperature (°C)

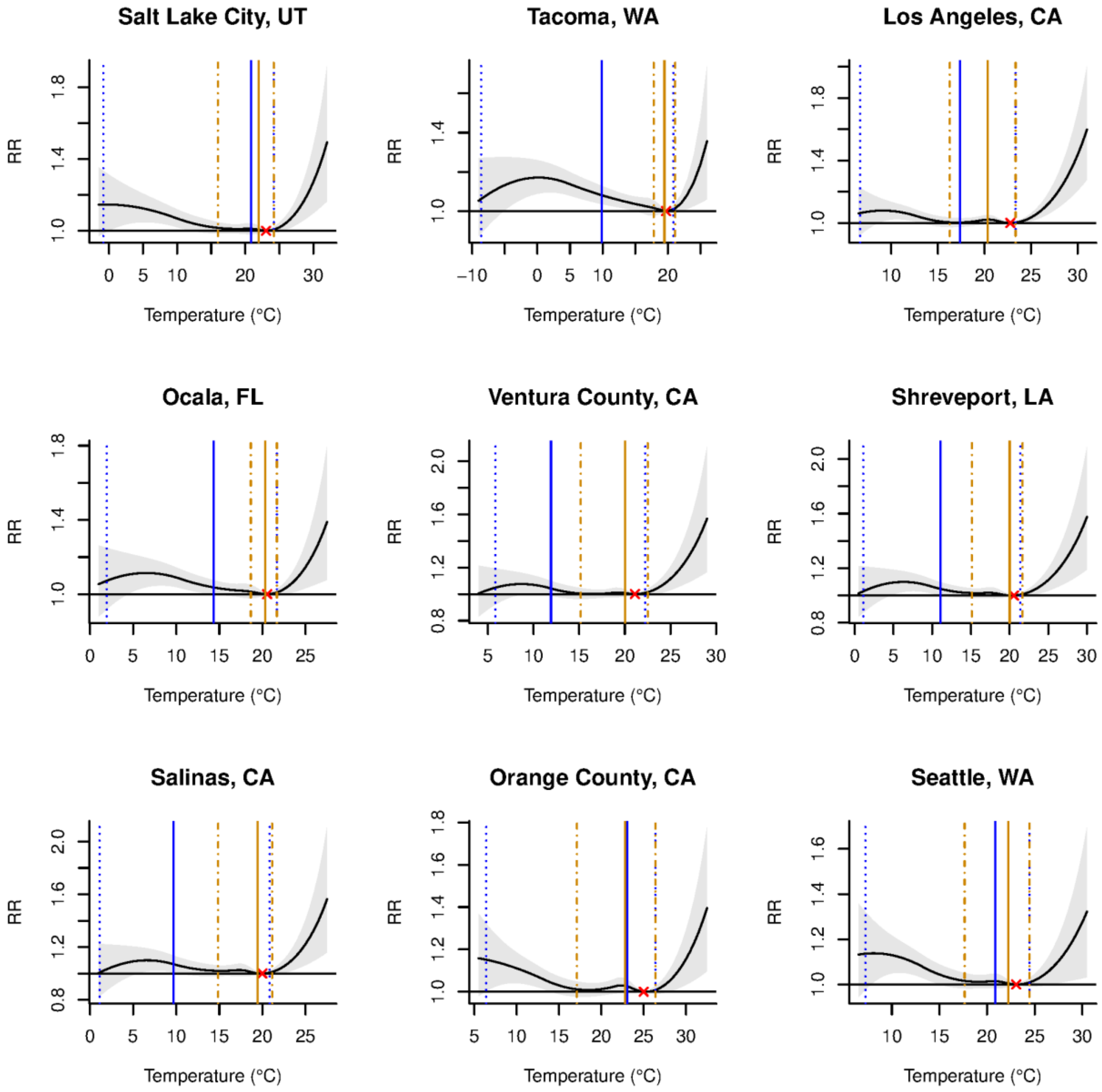

Salt Lake City, UT
RR
1.0 1.4 1.8
0 5 10 20 30
Temperature (°C)
Tacoma, WA
RR
1.0 1.4
−10 0 5 10 20
Temperature (°C)
Los Angeles, CA
RR
1.0 1.4 1.8
10 15 20 25 30
Temperature (°C)
Ocala, FL
RR
1.0 1.4 1.8
0 5 10 15 20 25
Temperature (°C)
Ventura County, CA
RR
0.8 1.2 1.6 2.0
5 10 15 20 25 30
Temperature (°C)
Shreveport, LA
RR
0.8 1.2 1.6 2.0
0 5 10 15 20 25 30
Temperature (°C)
Salinas, CA
RR
0.8 1.2 1.6 2.0
0 5 10 15 20 25
Temperature (°C)
Orange County, CA
RR
1.0 1.2 1.4 1.6 1.8
5 10 15 20 25 30
Temperature (°C)
Seattle, WA
RR
1.0 1.2 1.4 1.6
10 15 20 25 30
Temperature (°C)

Table S1. Descriptive Statistics for mortality and temperature in the 135 cities of the United States.

Cities	Total Death Count	Temperature Distribution (^oC)				
		Min	25%	Median	75%	Max
Akron, OH	107392	-26.7	2.2	10.8	18.9	30.8
Albuquerque, NM	73279	-14.7	6.4	14.4	22.5	32.2
Allentown-Bethlehem, PA	61366	-20.6	3.1	11.4	19.7	31.4
Atlanta, GA	310249	-15.0	10.6	18.1	24.4	32.5
Atlantic City, NJ	49410	-18.1	4.7	12.2	20.3	32.2
Austin, TX	69427	-8.6	15.0	21.9	27.8	35.0
Baltimore, MD	88852	-1.9	11.9	17.8	25.0	36.7
Bergen-Passaic, NJ	319591	-17.8	5.6	13.3	21.7	32.5
Bakersfield, CA	51337	-17.2	3.6	10.0	17.8	30.6
Boston, MA	239023	-18.6	5.3	13.3	21.7	34.7
Birmingham, AL	171109	-13.3	10.8	18.1	24.7	32.2
Barnstable-Yarmouth, MA	475683	-16.9	3.6	10.8	18.9	32.2
Baton Rouge, LA	62561	-8.6	14.4	21.4	26.7	32.8
Brownsville, TX	36059	-3.9	20.0	25.0	28.6	33.1
Buffalo, NY	212201	-21.1	1.4	9.7	18.3	30.3
Canton-Massillon, OH	77288	-26.7	2.2	10.8	18.9	30.8
Chattanooga, TN	49105	-20.6	5.8	14.2	21.4	31.7
Chicago, IL	82255	-12.5	9.4	16.9	23.9	32.5
Charleston, WV	60219	-15.8	9.2	16.9	23.9	32.8
Charlotte, NC	1115158	-26.7	1.7	10.6	19.4	33.6
Cincinnati, OH	171958	-23.6	5.0	13.6	21.4	33.1
Cleveland, OH	404057	-24.4	2.5	11.1	19.2	30.8
Columbus, OH	75994	-11.1	11.1	18.6	25.6	32.8
Columbia, SC	159353	-24.4	3.9	12.5	20.6	31.7
Dallas, TX	260718	-13.1	11.9	20.0	26.9	35.8
Dayton, OH	107272	-3.3	18.1	23.1	26.7	31.7
Denver, CO	108776	-27.5	3.3	12.2	20.3	32.2
Des Moines, IA	182600	-25.6	3.1	10.6	19.2	30.3
Detroit, MI	54488	-27.5	1.7	11.4	20.6	32.8
Dutchess County, NY	729077	-24.4	1.7	10.6	19.4	31.4
Daytona Beach, FL	43055	-20.3	1.9	10.3	18.3	31.7
El Paso, TX	73269	-8.6	10.8	18.9	25.6	36.7
Erie, PA	54723	-23.9	2.2	10.6	18.9	30.0
Flint, MI	75484	-24.4	0.6	9.2	18.1	30.8
Fresno, CA	104033	-2.8	11.7	17.8	24.7	38.6
Ft. Lauderdale, FL	308032	0.0	20.3	24.2	26.9	30.8
Fort Myers-Cape Coral, FL	88850	1.9	21.4	25.0	27.8	31.4

Fort Pierce-Port St. Lucie, FL	67004	-1.4	20.3	24.2	26.9	30.8
Fort Worth-Arlington, TX	172892	-7.5	12.2	20.0	26.7	35.6
Gary, IN	40680	-3.6	15.3	21.7	26.7	32.8
Galveston, TX	90669	-27.8	1.7	10.6	19.4	31.7
Greenville, SC	78804	-24.4	0.8	9.4	18.3	31.7
Greensboro, NC	65906	-14.2	7.8	15.6	22.5	30.6
Grand Rapids, MI	58344	-11.7	9.2	16.7	23.6	32.8
Hamilton, OH	49618	-23.6	5.0	13.6	21.4	33.1
Honolulu, HI	49992	-21.9	4.2	12.2	20.8	32.5
Houston, TX	159050	-18.3	2.2	10.6	19.0	31.1
Harrisburg-Carlisle, PA	75775	18.1	23.9	25.8	27.2	30.3
Hartford, CT	366340	-8.1	15.3	22.2	27.5	33.3
Indianapolis, IN	149459	-26.9	3.6	12.8	21.1	32.2
Jacksonville, FL	124017	-7.5	15.6	21.7	26.4	32.5
Jersey City, NJ	103084	-18.6	5.3	13.3	21.7	34.7
Kansas City, MO-KS	218933	-26.7	4.4	13.6	21.9	33.9
Knoxville, TN	80418	-21.1	8.1	15.8	23.1	30.3
Lubbock, TX	95395	-0.8	20.0	24.2	27.5	32.2
Lakeland-Winter Haven, FL	80724	-19.7	3.6	11.9	20.3	31.1
Lancaster, PA	37393	-23.3	0.6	9.2	17.8	30.8
Lansing, MI	182220	-5.6	11.9	20.0	29.2	41.1
Los Angeles, CA	1239036	6.1	14.7	17.5	19.7	31.1
Louisville, KY	139347	-22.8	6.7	15.3	23.1	34.2
Little Rock, AR	63901	-14.2	9.7	18.1	25.0	35.3
Las Vegas, NV-AZ	35407	-14.4	8.9	16.9	24.2	35.6
Madison, WI	48763	-28.9	0.0	8.9	18.1	32.8
McAllen-Edinburg-Mission, TX	49998	-3.6	20.3	26.1	29.4	36.7
Middlesex, NJ	88449	-0.3	19.4	23.6	26.7	31.9
Miami, FL	152003	-16.7	10.0	18.3	25.6	33.9
Milwaukee, WI	372130	3.3	23.1	25.8	28.1	31.4
Minneapolis-St. Paul, MN	110324	-17.5	3.9	11.9	20.0	32.5
Melbourne-Titusville-Palm Bay, FL	232056	-27.2	1.1	9.2	18.3	33.9
Memphis, TN	241475	-31.4	-1.4	8.9	18.9	32.5
Monmouth-Ocean, NJ	72746	-9.4	14.4	20.8	26.1	32.8
Mobile, AL	235036	-17.2	4.4	12.2	20.3	31.9
Myrtle Beach, SC	30268	-8.3	11.9	18.6	24.7	33.3
Naples, FL	36951	3.6	21.4	24.7	27.8	30.8
Nashua, NH	51115	-20.3	0.8	8.9	17.5	30.0
Nashville, TN	97358	-20.3	8.3	16.4	23.9	32.5
Nassau-Suffolk, NY	460192	-16.4	4.2	11.4	19.4	32.2
Newark, NJ	220980	-18.6	5.3	13.3	21.7	34.7

Newburgh, NY	49890	-19.7	2.2	10.6	18.9	30.8
New Haven-Meriden, CT	157415	-18.3	2.2	10.6	19.0	31.1
New London, CT	40419	-16.9	3.3	10.8	18.9	31.1
New York, NY	1367085	-16.4	5.8	13.3	21.7	34.4
Ocala, FL	325028	0.6	12.8	15.3	17.5	27.8
Oklahoma City, OK	58345	-3.6	18.1	23.1	26.7	32.2
Oakland, CA	118753	-17.8	8.3	16.7	24.2	34.7
Omaha, NE	71558	-26.7	1.9	11.9	20.8	33.3
Orange County, CA	320343	5.3	15.6	18.9	21.7	32.8
Orlando, FL	157019	-2.5	19.4	23.9	27.2	32.2
Pensacola, FL	50546	-9.2	15.0	21.4	26.7	34.2
Philadelphia, PA-NJ	911888	-17.5	5.6	13.6	21.9	33.3
Phoenix, AZ	386802	2.5	16.4	24.2	31.9	41.4
Providence-Fall River, RI-MA	317935	-25.0	3.1	11.9	19.4	30.3
Portland, ME	46217	-20.3	0.6	8.1	16.7	29.7
Portland, OR	210301	-11.1	7.5	12.2	17.8	29.7
Pittsburgh, PA	36108	-16.7	3.6	10.8	19.2	31.4
Punta Gorda, FL	37773	3.6	20.6	24.4	27.5	32.2
Raleigh, NC	58561	-15.6	8.6	16.4	23.3	32.2
Rochester, NY	72337	-15.6	8.6	16.4	23.3	32.2
Reading, PA	433285	3.6	14.4	18.9	23.9	36.7
Riverside-San Bernardino, CA	127040	-21.1	1.1	9.4	18.1	30.3
Rockford, IL	46380	-28.3	0.8	10.3	19.2	31.7
Sacramento, CA	172136	-2.5	11.1	16.4	21.7	34.7
Saginaw, MI	39515	-23.9	0.3	8.9	18.1	31.1
Salinas, CA	45929	0.6	11.9	14.7	16.9	27.8
San Antonio, TX	89770	-20.0	3.6	11.4	20.8	32.8
Sarasota-Bradenton, FL	186461	-7.8	15.0	22.2	27.8	34.2
Scranton--Wilkes-Barre--Hazleton, PA	151551	0.8	20.0	24.2	27.5	32.8
San Diego, CA	150119	-22.2	2.2	10.6	18.6	31.9
Seattle, WA	369956	6.4	15.0	17.8	20.3	30.8
San Francisco, CA	225451	-9.7	6.9	11.1	15.8	28.1
Shreveport, LA	248607	0.3	11.9	14.7	16.9	30.0
San Jose, CA	51716	-10.0	12.5	20.0	26.4	34.2
Salt Lake City, UT	176066	-1.9	12.5	16.4	20.0	32.2
Spokane, WA	68681	-23.6	1.9	8.3	16.1	30.6
Springfield, MA	94971	-18.3	2.2	10.6	19.0	31.1
Stockton-Lodi, CA	142216	-16.1	3.9	11.4	19.7	30.8
St. Louis, MO-IL	312923	-22.8	5.6	15.0	23.3	33.6
Stamford-Norwalk, CT	82225	-3.3	11.1	16.7	22.2	36.9
Syracuse, NY	84451	-23.6	1.1	9.7	18.3	30.3

Tacoma, WA	96086	-9.4	7.5	11.7	16.4	26.7
Tampa-St. Petersburg-Clearwater, FL	158555	-1.1	19.4	24.2	27.5	31.7
Toledo, OH	92004	-25.0	1.9	10.8	19.4	31.7
Trenton, NJ	58430	-16.4	4.2	11.9	20.0	31.7
Tucson, AZ	131053	-1.4	14.4	21.4	28.3	37.2
Tulsa, OK	95475	-17.5	8.3	16.9	24.7	35.3
Utica-Rome, NY	53724	-25.8	0.3	8.9	17.5	28.9
Ventura County, CA	87603	3.6	13.9	16.4	18.6	29.2
Virginia Beach, VA	187233	-15.0	8.9	16.4	23.6	33.1
Washington, DC-MD-VA	141028	-16.7	6.9	14.7	23.1	33.9
Wichita, KS	68542	-22.2	5.6	14.4	23.3	33.9
Wilmington, DE	76254	-18.9	5.0	12.8	21.1	31.9
West Palm Beach-Boca Raton, FL	135785	-21.1	1.1	9.2	17.5	28.9
Worcester, MA	233887	1.7	21.9	25.3	27.5	31.1
Youngstown-Warren, OH	62767	-22.2	4.2	12.8	20.8	31.1
York, PA	86656	-25.6	1.9	10.3	18.1	30.6
